# Digging the diversity of Iberian bait worms *Marphysa* (Annelida, Eunicidae)

**DOI:** 10.1371/journal.pone.0226749

**Published:** 2020-01-22

**Authors:** Daniel Martin, João Gil, Joana Zanol, Miguel A. Meca, Rocío Pérez Portela

**Affiliations:** 1 Center for Advanced Studies of Blanes (CEAB–CSIC), Blanes, Catalunya, Spain; 2 Centre of Marine Sciences, CCMAR, University of Algarve, Campus de Gambelas, Faro, Portugal; 3 Universidade Federal do Rio de Janeiro, Museu Nacional, Departamento de Invertebrados, Rio de Janeiro, Brazil; 4 Department of Natural History, University Museum of Bergen, University of Bergen, Bergen, Norway; 5 Rosenstiel School of Marine & Atmospheric Science, University of Miami, Miami, Florida, United States of America; CIIMAR Interdisciplinary Centre of Marine and Environmental Research of the University of Porto, PORTUGAL

## Abstract

During a visit to polychaete–rearing facilities in the vicinity of Bay of Cádiz (SW Iberian Peninsula, Atlantic Ocean), we sampled two populations of *Marphysa* (Annelida, Eunicidae) originally occurring at nearby intertidal soft bottoms, one being more than twice as long as the other at the same age. We analysed them using partial sequences of two mitochondrial genes, 16S rDNA and Cytochrome Oxidase I, and classical morphological observations. Our molecular results confirmed that the two populations corresponded to two different species, with PTP species delimitation values ranging from 0.973 (long–bodied species) to 0.999 (short–bodied species). Morphologically, the short–bodied species resembles the recently redescribed *M*. *sanguinea* (Montagu, 1813), but differs mainly in having some parapodia with two subacicular hooks (one bidentate and one unidentate) and three types of pectinate chaetae, Two isodont present all along the body, and one particularly large anodont asymmetric appearing only from mid–posterior parapodia. The long–bodied species resembles *Marphysa aegypti* Elgetany, El-Ghobashy, Ghoneim and Struck, 2018 both in size and in having very robust, unidentate subacicular hooks (single in most parapodia, two–both similar in size and form–in some posterior parapodia), but differs, among other features, in the maxillary formula, the number of acicula per parapodia and the number and shape of pectinate chaetae. Accordingly, we are here fully illustrating and formally describing the two Iberian populations as *Marphysa gaditana* sp. nov. (short–bodied) and *Marphysa chirigota* sp. nov. (long–bodied) and we are emending the description of *M*. *aegypti* based on our revision of the type material. Also, we discuss on the distribution of the species of the *sanguinea*–group and on the relevancy of taxonomically robust studies when dealing with species of commercial interest having the potential of being globally spread through human activities, as well as on the misunderstandings caused by the incorrect use of the “cosmopolitan species” concept.

## Introduction

In addition to the intrinsic interest of the annelid polychaetes as ubiquitous and highly abundant members of virtually all marine benthic ecosystems, some of them are increasingly exploited commercially. There is a growing demand of these organisms as fishing baits and, thus, they are being harvested all around the world as an integral part of global coastal life [1, 2], including Europe [3–5]. They have also been introduced in integrated polycultures to contribute managing organic matter and wastes produced by bivalves and fish [6], and are a natural source of proteins and omega–3 fatty acids so that they can be used as a nutritional resource and maturation diet in crustacean and fish aquaculture [1, 7, 8]. Therefore, in addition to the impacts of traditional bait harvesting [9], new mechanized methods of collection are being developed to increase efficiency, productivity and revenue [10, 11]. Thus, we may only expect further impacts and native habitat deterioration, as well as a greater pressure on their wild stocks. Importing allochthonous species is a well–established alternative to exploiting local populations. However, this transfers the harvesting impact to the often remote areas where baits are collected. Also, this may lead to accidental introductions (even to invasions) whether some viable specimens manage to escape from fishing hooks or are directly released into the wild by anglers at the end of their fishing journey [12, 13]. A more environmentally friendly alternative is rearing autochthonous species, although the number of feasible initiatives is still very low (e.g., [4, 14]). Nevertheless, these activities may also entail the destruction of local habitats either when implementing the facilities or during the routine functioning activities. On the other hand, culturing allochthonous species must be disregarded and discouraged due to the implicit risks of accidental releasing of living specimens that would directly impact the wild surroundings.

So far, no commercial polychaete aquaculture initiatives have been successfully implemented in the Iberian Peninsula. There were some attempts by researchers from the “Grupo de Ecología” of the “Universidad de Cantabria”, in cooperation with the company TEICAN Mediambiental SL. Also, the Institute of Marine Sciences of Andalucía (ICMAN), in cooperation with the private companies “Comercial de Cebos para la Pesca S.L” and SEAPRTNERS, attempted to develop cultures of supposedly *Marphysa sanguinea* (Montagu, 1813) [15] based on their relatively abundant, autochthonous populations in the Bay of Cádiz (SW Atlantic coasts of the Iberian Peninsula) [16–18]. The present work resulted from a visit of DM and JG to the facilities built during an initial phase of the project developed at the Bay of Cádiz.

The genus *Marphysa* Quatrefages, 1866 [19] is a typical Eunicidae (Annelida, Eunicida), a family that currently comprises 71 nominal species [20]. They are free–living, tubicolous or burrowing polychaetes inhabiting a wide range of habitats, from soft sediments to rocky grounds, typically in warm and temperate waters. Bathymetrically, they occur mainly from intertidal to shallow subtidal depths, while the few species described from shelf to bathyal depths are generally poorly known and thus need further revision. Many intertidal species are a valuable biological and economical resource, widely used and highly appreciated as fishing baits for many decades in the Iberian Peninsula [13], but also elsewhere [3, 21–34], being commonly known with the vernacular names of “rosca” or “gusana de sangre” in Spanish, or “blood worm”, “rock worm” or “clam worm” in English. This includes the type species, *M*. *sanguinea*, originally described from Devon, UK and recently redescribed based on a neotype from a nearby locality [29, 35, 36].

During our visit to the polychaete–rearing facilities at Bay of Cádiz, we realised that the native populations of *Marphysa* originally collected from nearby intertidal soft-bottoms and designated by local fishermen as “sand” and “mud” *Marphysa* (according to their original habitats), clearly represented two different morphotypes. In spite of having being reared under the same environmental conditions, the adults of the former were more than twice longer and much more active than the latter at the same age.

We hypothesized initially that the short–bodied population corresponded to *M*. *sanguinea*, which has been widely reported in the Iberian Peninsula [37], while the other could represent an undescribed species. To resolve this question, we analysed specimens from both populations by using partial sequences of the mitochondrial genes 16S rDNA (hereafter 16S) and Cytochrome Oxidase I (COI), as well as classical morphological observations. As a result, both south Iberian morphotypes are here described as species new to science. Both species are compared with the most similar ones, including *Marphysa aegypti* Elgetany, El-Ghobashy, Ghoneim and Struck, 2018 [31], whose description is emended based on our revision of the type material. We finally discuss on the distribution of the species of the *sanguinea*–group and on the relevancy of taxonomically robust studies when dealing with species of commercial interest having the potential of being spread globally.

## Material and methods

### Collection

Samples were originally collected by professional fishermen in intertidal shores of the Natural Park of the Bay of Cádiz (SW Iberian Peninsula) and transported to isolated polychaete–rearing facilities located at the San Ramón saltworks (Chiclana de la Frontera, Spain). Within the frame of an agreement between “Comercial de Cebos para la Pesca” and the Institute of Marine Sciences of Andalucía (ICMAN-CSIC), the specimens studied herein were collected by hand digging at the rearing facilities on the 10^th^ of May 2011. For morphological observations, the specimens were gently relaxed prior to being fixed in a buffered 10% seawater/formaldehyde solution and then transferred to 70% ethanol. For molecular purposes, fragments of the specimens obtained by natural autotomy of posterior ends were directly preserved in absolute ethanol and kept in the dark at -20°C.

### DNA extraction, amplification and sequencing

Total DNA was extracted from small body wall pieces using REDExtract–N–Amp kit (Sigma Aldrich, www.sigma.com) and DNAeasy Tissue Kit (Qiagen) for 16S and COI genes, respectively, following the manufacturer’s protocol. REDExtract–N–Amp kit DNA extractions were diluted (1/6) in ultrapure Millipore water before using them for PCR amplification of fragments of the mitochondrial gene 16S. We amplified 826 bp of 16S and 660–700 bp of COI. We used primers designed in this study with the software Primer3 v 0.4.0 [38] for the 16S: Mar_16SF 5’ GTGAGCTGATCTTTACTTGC 3’ and Mar_16SR 5’ GCTCTGGAGGAAGATTAGTC 3’. For COI, we used the primers polyLCO 5’ GAYTATWTTCAACAAATCATAAAGATATTGG 3’ and polyHCO 5’ TAMACTTCWGGGTGACCAAARAATCA 3’ [39]. For 16S, PCR amplification reactions were performed in a 20 μL total reaction volume with 10 μL of REDEXtract–N–ampl PCR reaction mix (Sigma Aldrich), 0.8 μL of each primer (10 μM), 7.4 μL of ultrapure water, and 1 μL of DNA diluted template in the case of 16S. For COI PCR reactions were performed in a 25 μL total reaction volume with 2.5 μL of NH_4_ No MgCl_2_ Bioline Reaction Buffer (10X), 0.5 μL of MgCl_2_ (50 mM), 1 μL of nucleotide mix (10 mM each dNTP), 0.8 μL of each primer (10 μM), 0.15 μL of BIOTAQ DNA polymerase (5 U/μL, Bioline), 1 μL of template DNA and 17.25 μL of nuclease–free water. The PCR temperature profile for 16S was as follows: a first step at 95°C for 5 min, followed by 35 cycles at 94°C for 1 min + 42°C for 1 min + 72°C for 1 min, and a final step of 72°C for 5 min. For COI: a first step at 94°C for 10 min, followed by 5 cycles at 94°C for 40 sec + 44°C for 40 sec + 72°C for 1 min, 35 cycles at 94°C for 40 sec + 51°C for 40 sec + 72°C for 1 min, and a final step of 72°C for 5 min. Agarose gel electrophoresis were used to visualise PCR products and to confirm fragment amplifications. Successful amplifications were purified and sequenced in both directions (forward and reverse) by Macrogen, Inc. (Seoul, Korea) with the same primers used in amplifications.

Additional sequences belonging to other *Marphysa* species, together with other genera of Eunicidae and one Onuphidae, were obtained from GenBank (Table 1). Sequences of 16S rDNA were edited using Geneious vs. R8 and aligned along with GenBank sequences using the Q–INS–I option of MAFFT v.7 [40] and manually adjusted. COI sequences were edited using BioEdit v. 7.0.5.3 software [41], translated into aminoacids and aligned by hand together with GenBank additional sequences in Mesquite v.3.6 [42].

**Table 1:** Species and sequences included in the molecular analyses.

			GenBank Accession Number	
Family	Species	Locality	COI	16S rDNA
Eunicidae	*M*. *chirigota* sp. nov.	Cádiz Bay, SW Iberian Peninsula	MN816441	MN813670
			MN816442	MN813671
			MN816443	MN813672
	*M*. *gaditana* sp. nov.	Cádiz Bay, SW Iberian Peninsula	MN816444	MN813673
			Non available	MN813674
	*M*. *aegypti* Elgetany, El-Ghobashy, Ghoneim and Struck, 2018 [31]	Al ferdan, Suez Canal	MF196968 [31]	Non available
		off Alexandria, Mediterranean Sea	MF196969, MF196970, MF196971 [31]	Non available
	*M*. *bifurcata* Kott, 1951 [43]	Northeast Australia	KX172177, KX172178 [44]	Non available
	*M*. *brevitentaculata* Treadwell, 1921 [45]	Quintana Roo, México	GQ497548 [46]	GQ478158 [46]
	*M*. *californica* Moore, 1909 [47]	California, USA	GQ497552 [46]	GQ478162 [46]
	*M*. *corallina* (Kinberg, 1865) [48]	South Africa	KT823271, KT823300, KT823306, KT823343, KT823371, KT823389, KT823410 [49]	Non available
	*M*. *fauchaldi* Glasby & Hutchings, 2010 [50]	North Australia	KX172165 [44]	Non available
	*M*. *hongkongensa* Wang, Zhang & Qiu, 2018 [51]	Tolo Harbour, Hong Kong	MH598525 [51]	MH598527 [51]
			MH598526 [51]	MH598528 [51]
	*M*. *iloiloensis* Glasby, Mandario, Burghardt, Kupriyanova, Gunton & Hutchings, 2019 [8]	Philippines	MN133418, MN106279, MN106280, MN106281 [8]	Non available
	*M*. *kristiani* Zanol, da Silva & Hutchings, 2016 [52]	Southeast Australia	KX172141, KX172142, KX172143, KX172144, KX172145, KX172146, KX172147, KX172148, KX172149, KX172150, KX172151, KX172155, KX172152, KX172153, KX172154, KX172156, KX172157, KX172158, KX172159, KX172160, KX172161, KX172162, KX172163 [52]	Non available
	*M*. *mullawa* Hutchings & Karageorgopoulos, 2003 [29]	East and Southeast Australia	KX172166, KX172167, KX172168, KX172169, KX172170, KX172171, KX172172, KX172173, KX172174, KX172175, KX172176 [52]	Non available
	*M*. *sanguinea* (Montagu, 1813) [15]	Callot Island, Northern Bretagne, France(locality corrected from that in [46])	GQ497547 [46]	GQ478157 [46]
		? [53]	Non available	AY838836 [53]
		Cornwal, UK	MK541904, MK950851, MK950852 [36]	Non available
		Arcachon, France	MK950853 [36]	Non available
		Brest, France	MK967470 [36]MN106282, MN106283, MN106284 [8]	Non available
	*M*. *mossambica* (Peters, 1854) [54]	Philippines	JX559751 [46]	JX559747 [46]
		Australia	KX172164 [52]	Non available
	*M*. *pseudosessiloa* Zanol, da Silva & Hutchings, 2017 [52]	Southeast Australia	KY605405, KY605406 [44]	Non available
	*M*. *regalis* Verrill, 1900 [55]	Ceará, Brazil	GQ497562 [46]	GQ478165 [46]
	*M*. *tripectinata* Liu, Hutchings & Sun, 2017 [33]	Beihai, China	MN106271, MN10622, MN1062723, MN106274, MN106275, MN106276, MN106277, MN106278 [33]	Non available
	*M*. *victori* Lavesque, Daffe, Bonifácio & Hutchings, 2017 [3]	Arcachon Bay, France	MG384996, MG384999	Non available
			MG384997 [3]	MG385000 [3]
			MG384998 [3]	MG385001 [3]
	*M*. *viridis* Treadwell, 1917 [56]	Ceará, Brazil	GQ497553 [46]	GQ478163 [46]
	*Marphysa* sp.	Sado Estuary, Portugal	KR916870 [57]	Non available
		Cap de la Hague, France^1^	AY040708 [58]	Non available
		Sado Estuary, Portugal^1^	KR916871, KR916872, KR916873 [57]	Non available
		Eastern Shore, Virginia, USA^1^	KP254223, KP254644 KP254890, KP255196 [59]	Non available
		Indian River Lagoon, Florida, USA^1^	KP254503, KP254537, KP254643, KP254743, KP254802 [59]	Non available
		China^1^	NC023124* [60]	NC023124* [60]
		China^1^	KF733802* [60]	KF733802* [60]
	*Paucibranchia bellii* (Audouin & Milne Edwards, 1833) [61] ^2^	Bay of Biscay, Spain	KT307661 [62]	Non available
		Banyuls, France	Non available	DQ779623 [63]
		?	Non available	AY838835 [53]
	*Paucibranchia disjuncta* (Hartman, 1961) [64]^2^	California, USA	GQ497549 [46]	GQ478159 [46]
	*Paucibranchia* sp. ^2^ [65]^3^	Philippines	JX559753 [66]	JX559750 [66]
	*Nicidion angeli* (Carrera-Parra and Salazar-Vallejo, 1998) [67]^2^	Carrie Bow Cay, Belize	Non available	GQ478161 [46]
	*Palola viridis* Gray *in* Stair, 1847 [68]	Kosrae, Micronesia	GQ497556 [46]	GQ478167 [46]
	*Eunice* cf. *violaceomaculata* Ehlers, 1887 [69]	Carrie Bow Cay, Belize	GQ497542 [46]	GQ478148 [46]
	*Leodice rubra* (Grube, 1856) [70]	Ceará, Brazil	GQ497528 [46]	GQ478132 [46]
Onuphidae	*Hyalinoecia* sp.	Massachusetts, USA	GQ497524 [46]	GQ478125 [46]

*Complete mitochondrial genome.

^1^Species identified as *M*. *sanguinea* in GenBank, but identification incorrect according to [3] and our study.

^2^Genus updated, species is under *Marphysa* in GenBank.

^3^This specimen belongs to the *Marphysa bellii* group (JZ, personal observation), which was recently considered to represent a different genus and described as *Paucibranchia*.

### Species delimitation

To explore the potential clustering of our samples to other *Marphysa* species, we reconstructed phylogenetic trees for both markers separately, including sequences of *Marphysa* available in GenBank (NCBI), of published studies or thesis that authors had access to, and of six outgroup taxa (five of other genera of Eunicidae and one Onuphidae) (Table 1). We used jModelTest 2 [71] as implemented in CIPRES Science Gateway V. 3.3 [72]. The most appropriate evolutionary models for our data determined by the Akaike Information Criterion (AIC) were GTR+I+G for 16S and HKY+I+G for COI. Bayesian Inference (BI) reconstructions were ran in MrBayes 3.2.6 [73] as implemented in CIPRES Science Gateway V. 3.3 [72], with two independent runs (each performed for four Markov–Chain Monte Carlo simulations) for 9 million generations for 16S and for COI analyses, sampled every 1,000 generations and initial 25% trees discarded as burning. We considered convergence of runs (average standard deviation ≤ 0.01) and effective sample size of parameters (ESS ≥ 200) calculated using Tracer v. 1.7.1 [74] to evaluate runs and accept results of the analyses. For both datasets, we calculated pairwise genetic distance using K2P model and partial gap deletion (cut-off 95%) in MEGAX [75].

The Poisson Tree Processes model (PTP, [76]) using BI rooted trees, 100,000 generations and removing all outgroups with exception of *Paucibranchia* was applied to infer putative species boundaries among our target samples and GenBank sequences using the webserver The Elexis Lab (https://sco.h-its.org/exelixis/web/software/PTP/index.html). We visually checked the convergence of MCMC runs in the maximum likelihood plot generated by the software.

### Morphological study

To describe the diagnostic morphological features, we followed the terminology proposed by [52, 67, 77]. When necessary for descriptions or photography, relevant morphological structures (e.g., jaw apparatus, parapodia) were dissected and mounted on slides. Particularly, we dissected representative parapodia of the new species from anterior (5), median (40) and posterior (120–130) chaetigers to illustrate parapodial morphology and along-body variability.

Whole body pictures were taken with a PowerShot–SX710–HS digital camera. Light microscopy photos were taken with a CMEX 5 digital camera connected to a ZEISS Stemi CS–2000–C stereomicroscope and with a SP100 KAF1400 digital camera connected to a Zeiss Axioplan compound microscope. When necessary, dissected structures were stained with Methyl blue to highlight relevant characters. The same equipment was used to measure relevant morphological structures (with the help of the ISListen software, version 5.4(1) copyright by Tucsen Photonics Co. Ltd.), as well as to make the drawings (with the help of the Adobe Illustrator CC, version 2015.3.1, and Photoshop CC, version 2015.5.1, copyright by Adobe systems Inc.).

For Scanning Electron Microscope (SEM) observations, specimens were prepared using standard SEM procedures [78]. SEM images were taken with a Hitachi TM3000 TABLETOP microscope at the SEM service of the CEAB–CSIC.

The type series of the Iberian populations are deposited at the Museo Nacional de Ciencias Naturales of Madrid (MNCN) and in the Natural History Museum Oslo (NHMO). The type material of *M*. *aegypti* was revised thanks to a kind loan of the NHMO.

### Nomenclatural acts

The electronic edition of this article conforms to the requirements of the amended International Code of Zoological Nomenclature, and hence the new names contained herein are available under that Code from the electronic edition of this article. This published work and the nomenclatural acts it contains have been registered in ZooBank, the online registration system for the ICZN. The ZooBank LSIDs (Life Science Identifiers) can be resolved and the associated information viewed through any standard web browser by appending the LSID to the prefix http://zoobank.org/. The LSID for this publication is: urn:lsid:zoobank.org:pub:5053C03E-0822-4581-8F7A-3FE78C6BC4EA. The electronic edition of this work was published in a journal with an ISSN, and has been archived and is available from the following digital repositories: PubMed Central, LOCKSS, ResearchGate and DigitalCSIC.

## Results

### Molecular analyses

**Fig 1:**
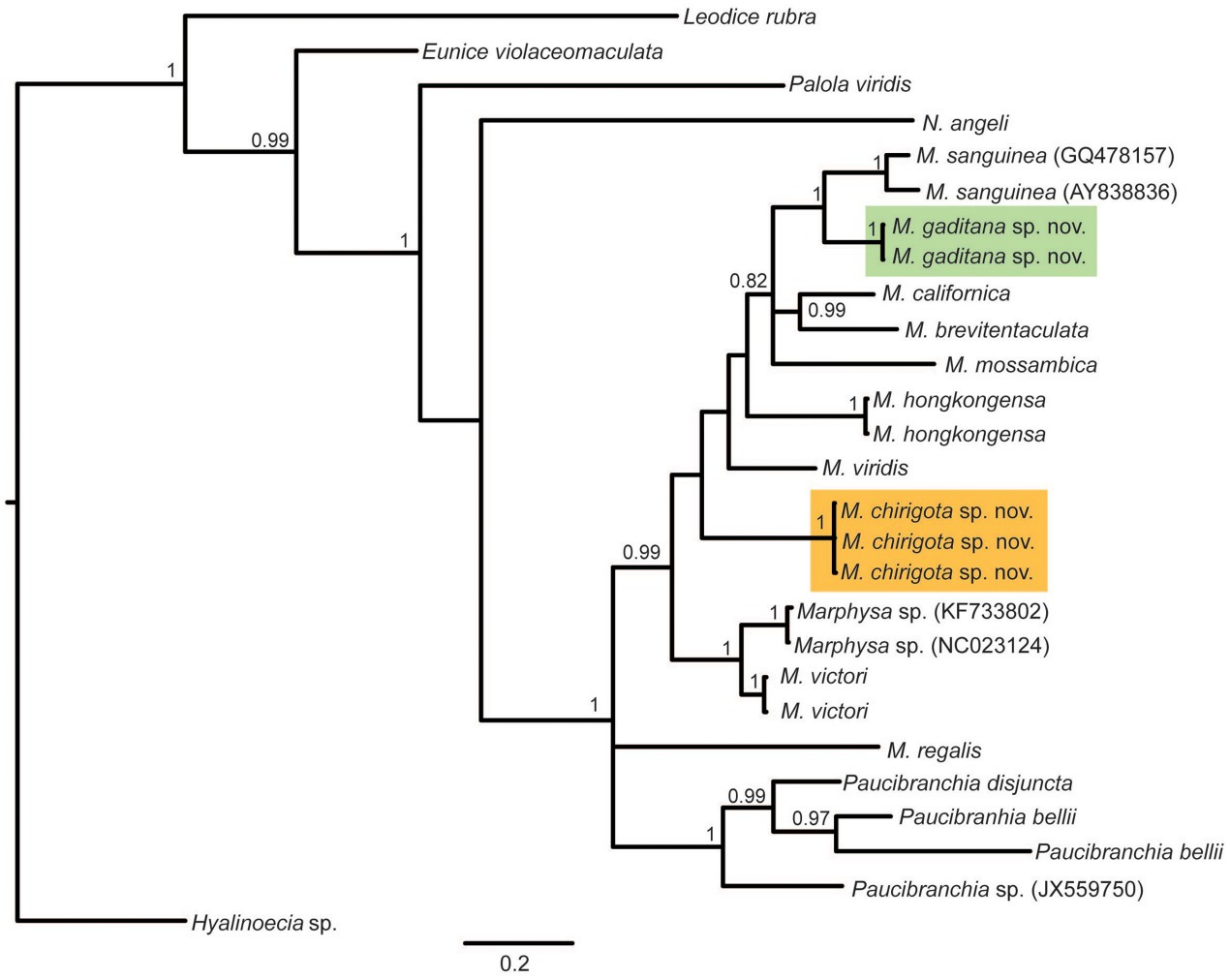
Bayesian Inference tree based on the 16S rDNA sequences. Posterior probability above 0.70 shown on branches. Orange and green clades correspond to the new species found in this study. Codes in parentheses are GenBank accession numbers.

**Fig 2:**
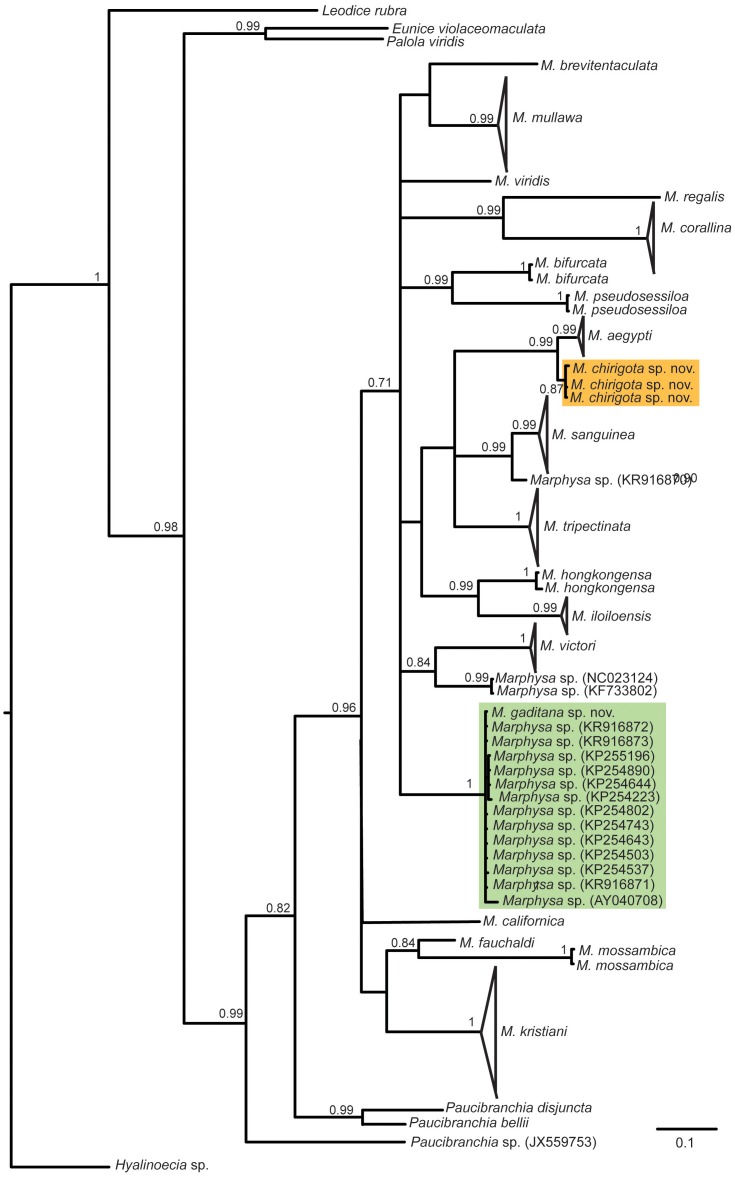
Bayesian Inference tree based on the COI sequences. Posterior probability above 0.70 shown on branches. Orange and green clades correspond to the new species found in this study. Codes in parentheses are GenBank accession numbers.

In resulting trees, specimens from Cádiz formed two different well supported monophyletic groups (Figs 1 and 2). The lowest 16S K2P pairwise distances between both focus species and other sequences available in the GenBank were, respectively, 9.2% between *M*. *gaditana* sp. nov. and *M*. *sanguinea* (AY838836) and 16.1% between *M*. *chirigota* sp. nov. and *M*. *californica* Moore, 1909 [47] (GQ478162). For COI sequences, the lowest K2P pairwise distances for *M*. *gaditana* sp. nov. were 0–1.9% with specimens from France, Portugal and East Coast of USA misidentified as *M*. *sanguinea* (Fig 2, green clade), while for *Marphysa chirigota* sp. nov., the lowest COI K2P pairwise distances were with *M*. *aegypti* (2.9–3.74%).

Results of PTP analyses supported that the two Iberian populations correspond to two different species, with 16S and COI species delimitation support values ranging, respectively, from 0.97 and 0.85 (*M*. *chirigota* sp. nov.) to 1 and 0.95 (*M*. *gaditana* sp. nov.) (full results as S1 File). *Marphysa chirigota* sp. nov. resulted in a species distinct from all others of the genus that have 16S and COI sequences available in GenBank, while *M*. *gaditana* sp. nov. formed the a single clade together with numerous specimens previously identified as *Marphysa sanguinea* and *Marphysa* sp. (Fig 2).

### Taxonomic account

Order Eunicida Dales, 1962 [79]

Family Eunicidae Berthold, 1827 [80]

Genus *Marphysa* Quatrefages, 1866 [19]

Type species: *Marphysa sanguinea* (Montagu, 1813) [15], by subsequent designation.

#### *Marphysa gaditana* Martin, Gil and Zanol sp. nov.

LSID: urn:lsid:zoobank.org:act:4F4A6736-A267-4B40-AF6D-205091235ACF Figs 3A, 3B, 4, 5A, 5B, 6, 7A, 7B, 8, 9A, 9B, 10A and 10D.

**Fig 3:**
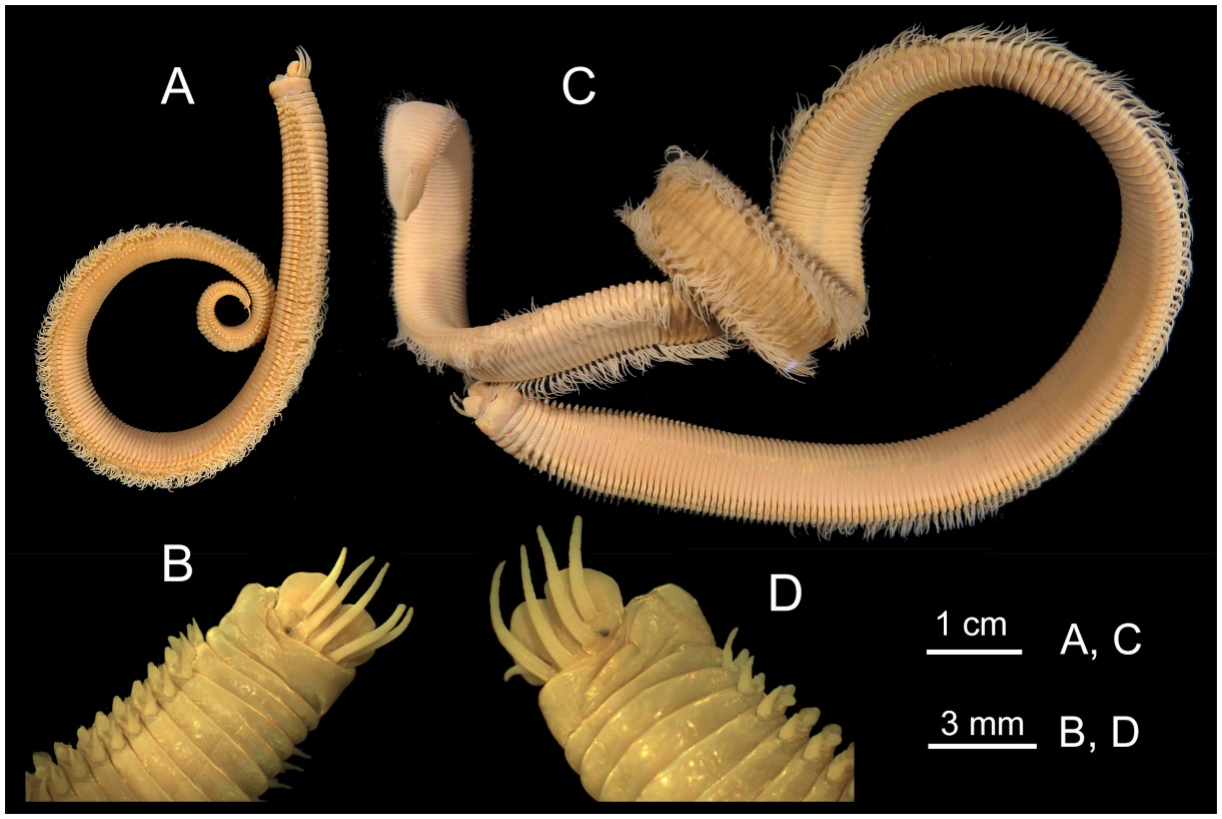
*Marphysa gaditana* sp. nov. A. Whole body. B. Detail of the anterior end showing the position of the eyespot. ***Marphysa chirigota* sp. nov**. C. Whole body. D. Detail of the anterior end showing the position of the eyespot.

**Fig 4:**
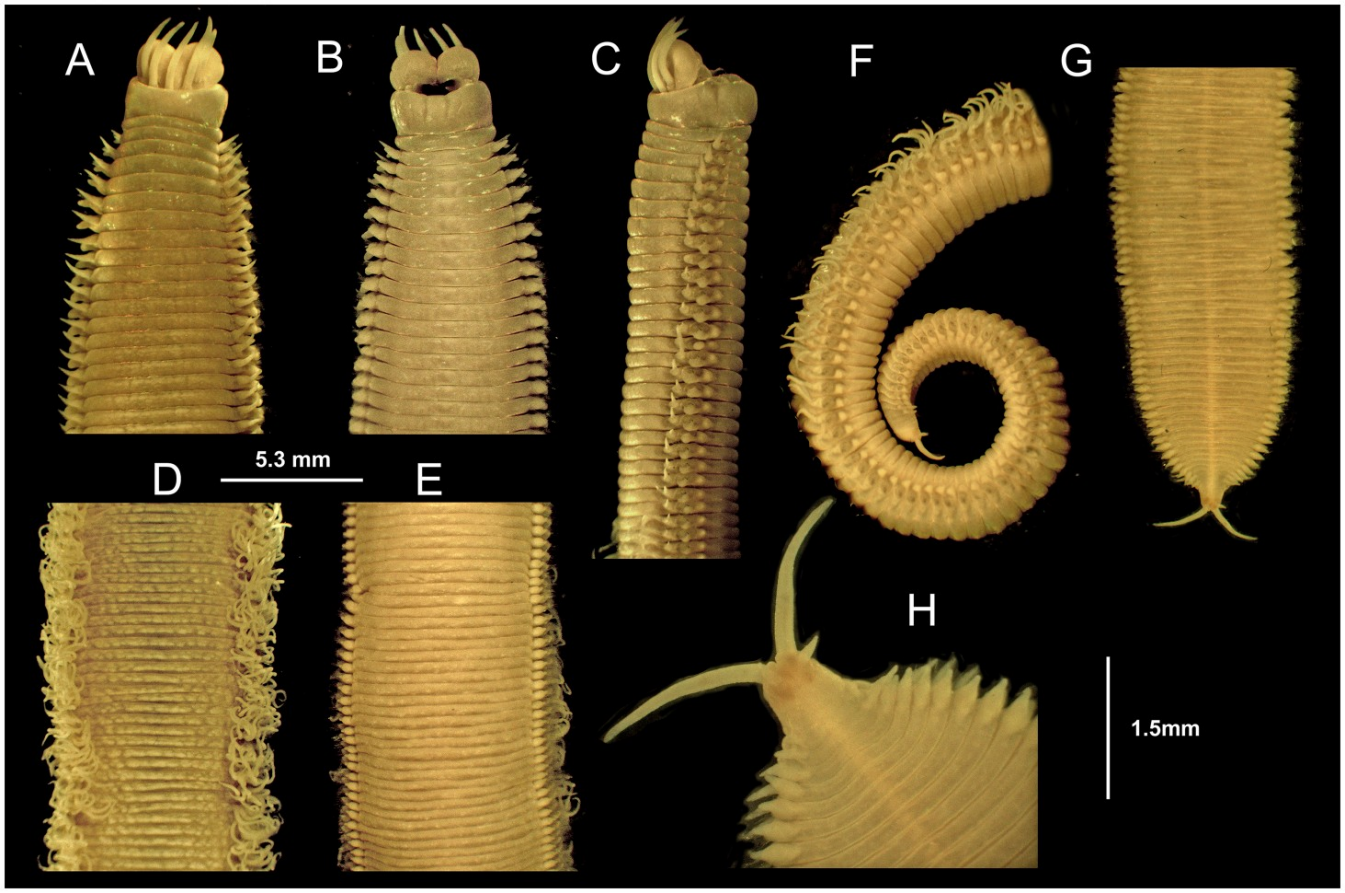
*Marphysa gaditana* sp. nov. Anterior end. A. Dorsal view. B. Ventral view. C. Lateral view. Mid-body. D. Dorsal view. E. Ventral view. Posterior end. F. Lateral view. G. Ventral view. H. Detail of pygidium showing the two pairs of anal cirri. A–G same scale.

**Fig 5:**
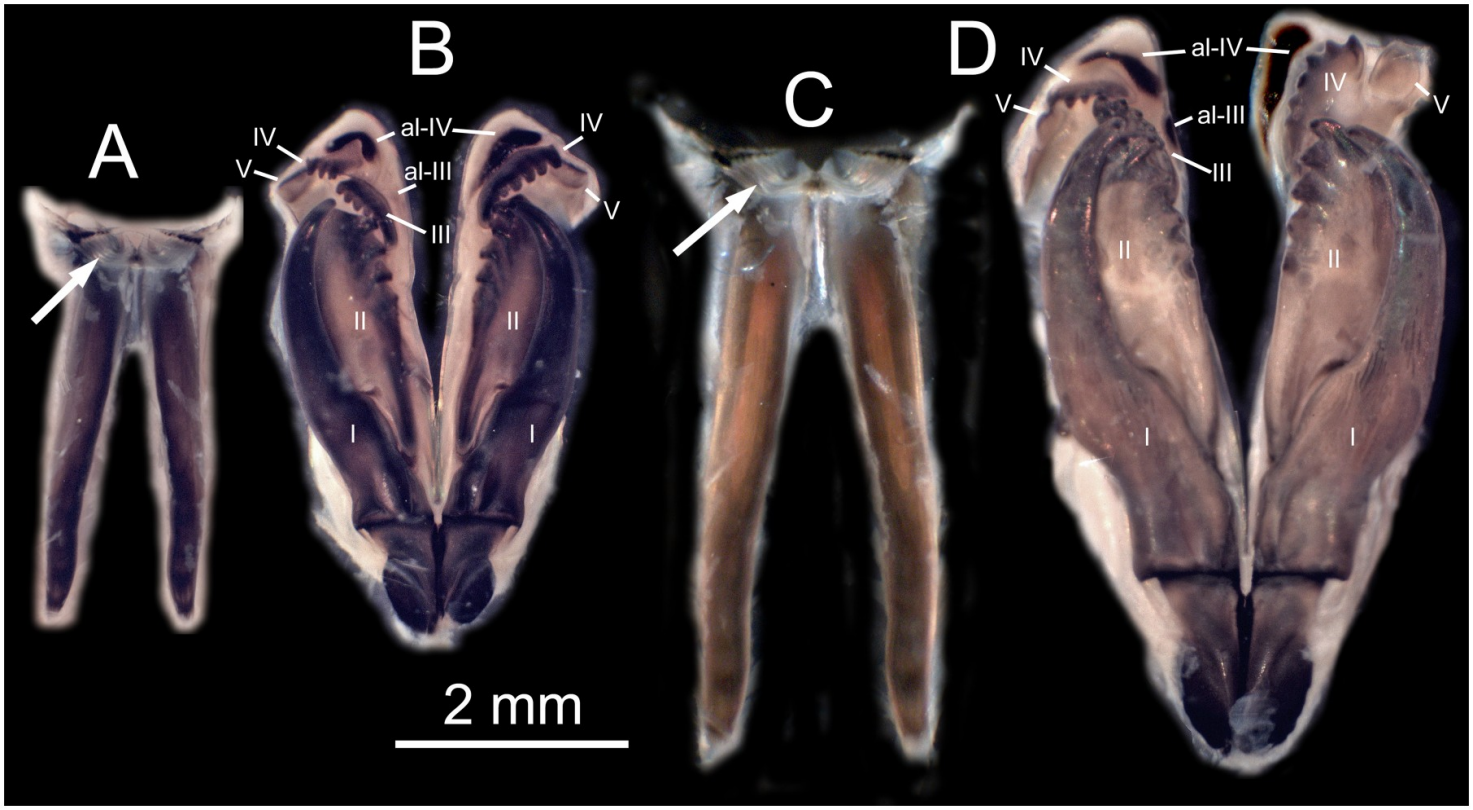
*Marphysa gaditana* sp. nov. A. Dissected mandible. B. Dissected maxillae. ***Marphysa chirigota* sp. nov**. C. Dissected mandible. D. Dissected maxillae. Arrows pointing on sclerotized matrix. Roman numerals: number of the maxilla; al: attachment lamella.

**Fig 6:**
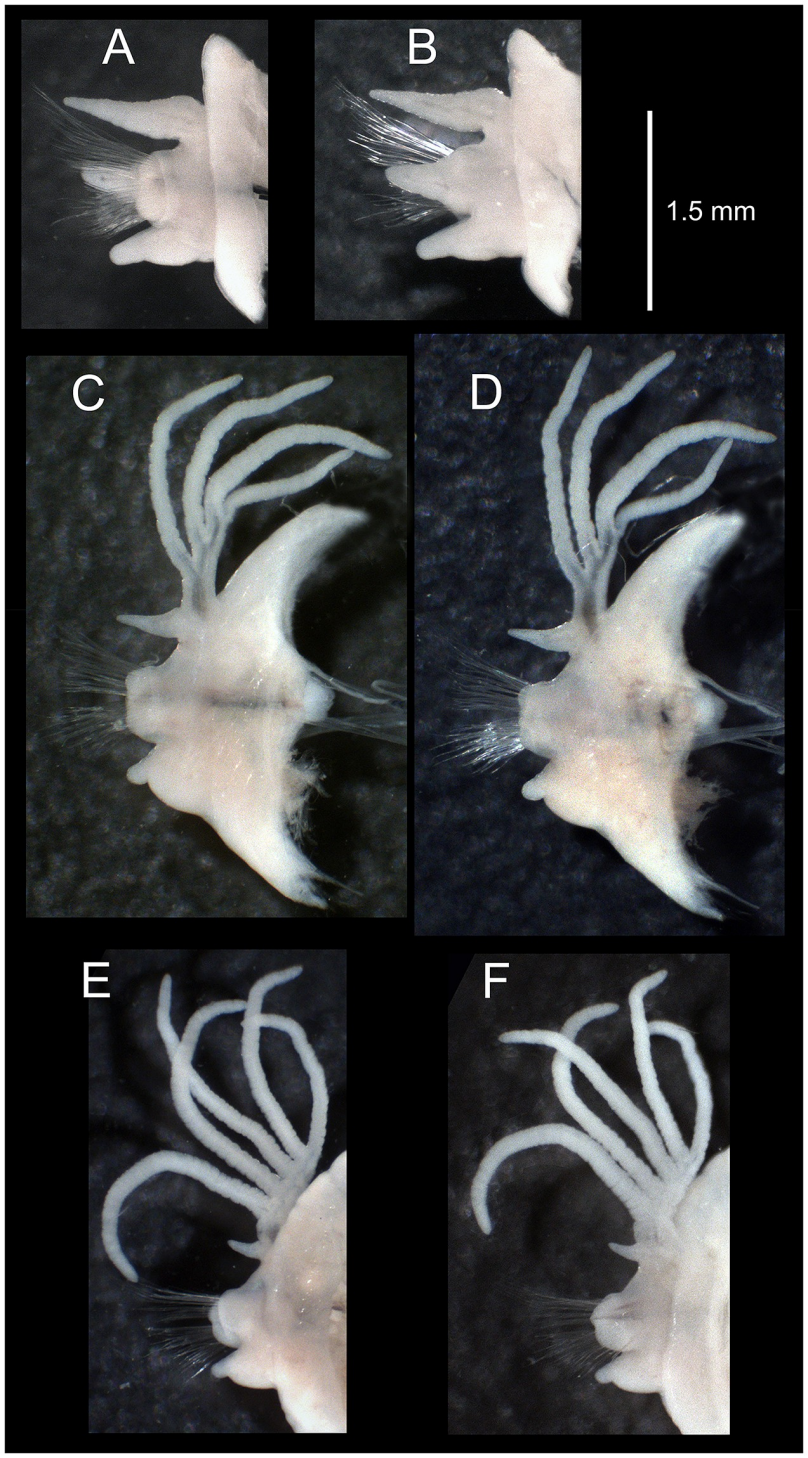
*Marphysa gaditana* sp. nov. Parapodium from chaetiger 5 in antero–posterior (A) and postero–anterior (B) views. Parapodium from chaetiger 40 in antero–posterior (C) and postero–anterior (D) views. Parapodium from a posterior branchial chaetiger (120) in antero–posterior (E) and postero–anterior (F) views.

**Fig 7:**
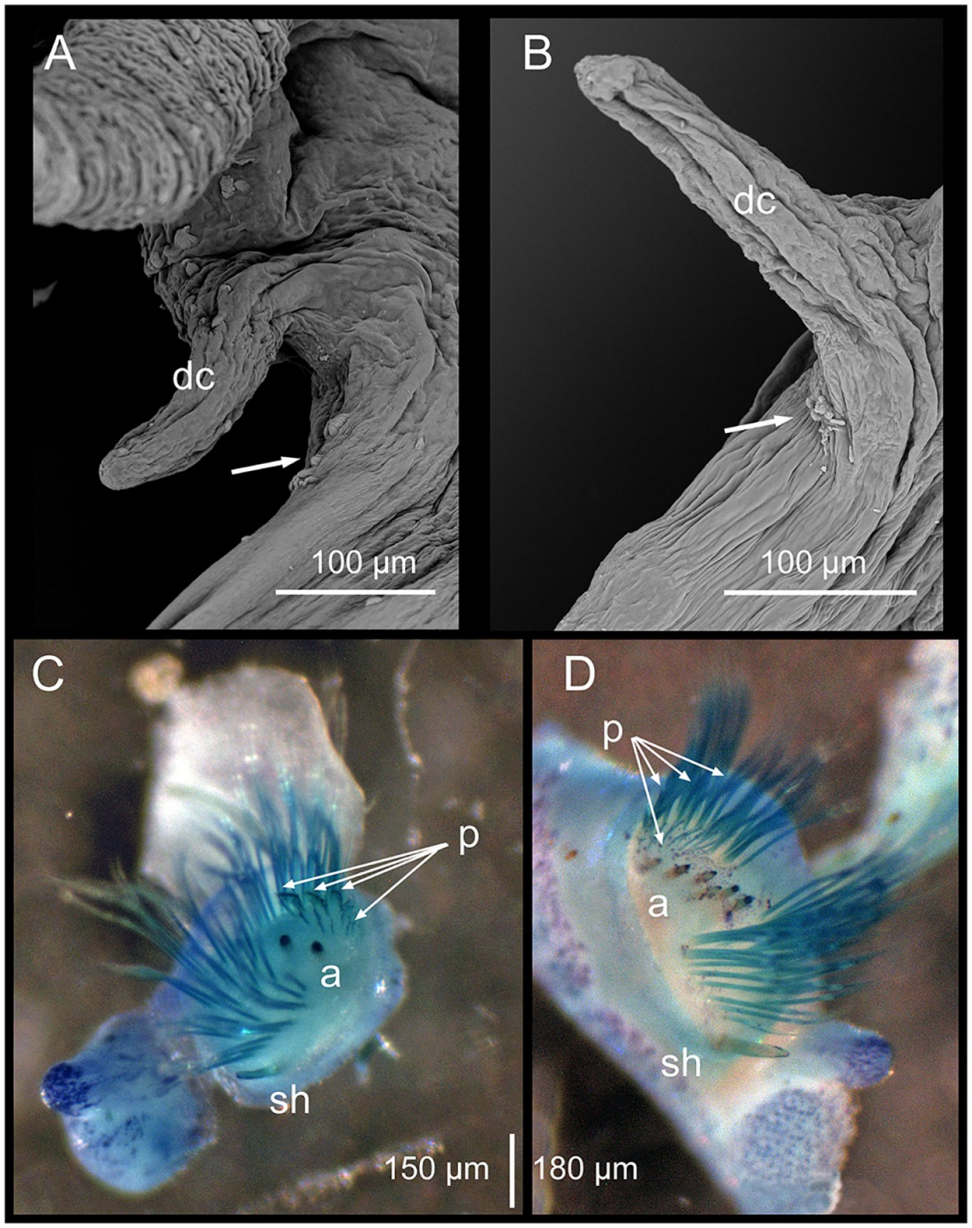
Position of lateral sense organs under SEM (A, B) and neurochaetae in parapodia stained with Methyl blue (C, D). A, C. *Marphysa gaditana* sp. nov. B, D. *Marphysa chirigota* sp. nov. White arrows: lateral sense organs; dc: dorsal cirri; p: pectinate chaetae; a: aciculae; sh: subacicular hooks.

**Fig 8:**
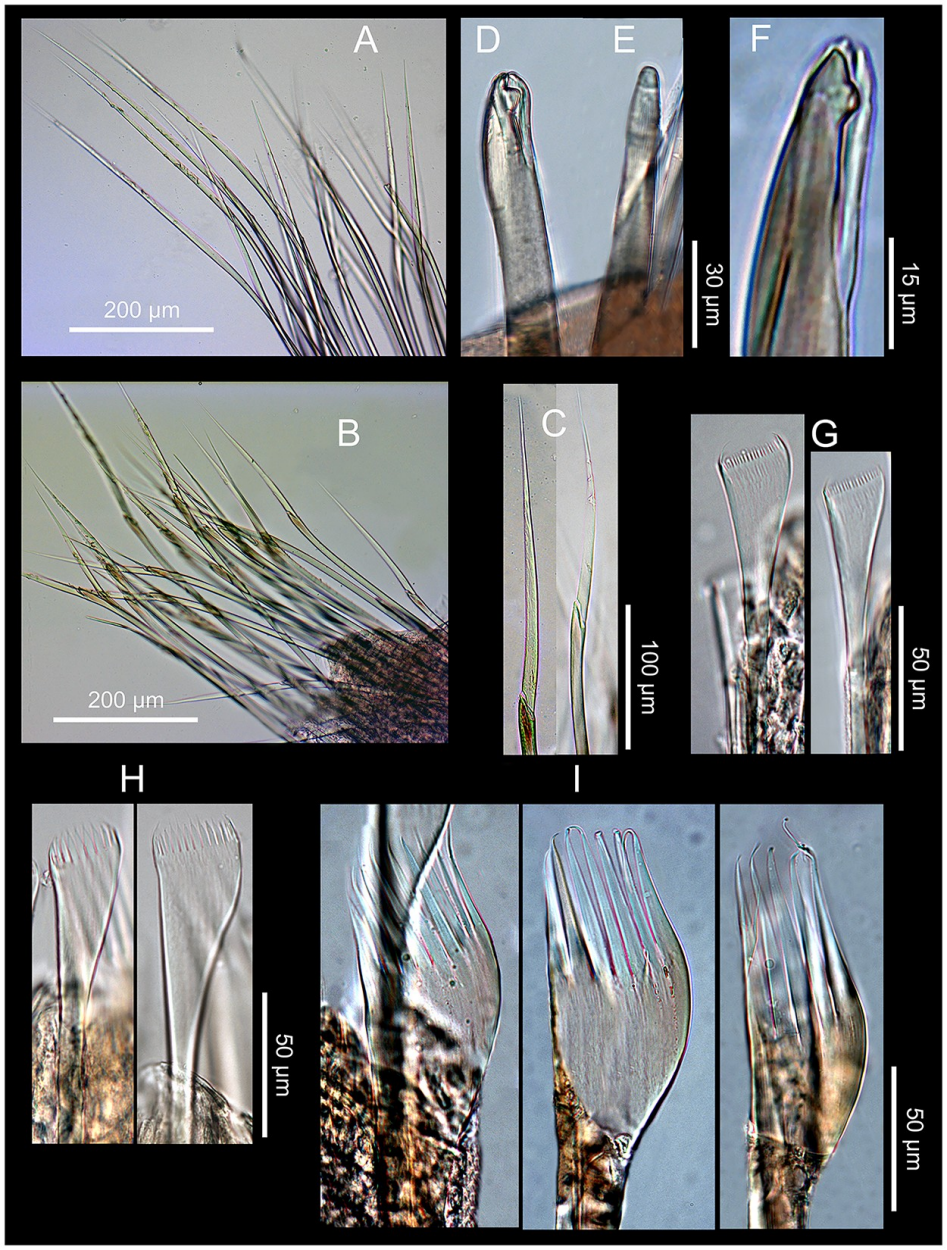
*Marphysa gaditana* sp. nov. A. Supracicular limbate chaetae. B. Subacicular spiniger compound chaetae. C. Detail of a spiniger compound chaeta. D. Bidentate subacicular hook. E. Unidentate subacicular hook. F. Detail of the tip of a bidentate acicular hook. G. Type 1 pectinate chaetae. H. Type 2 pectinate chaetae. I. Type 3 pectinate chaetae.

**Fig 9:**
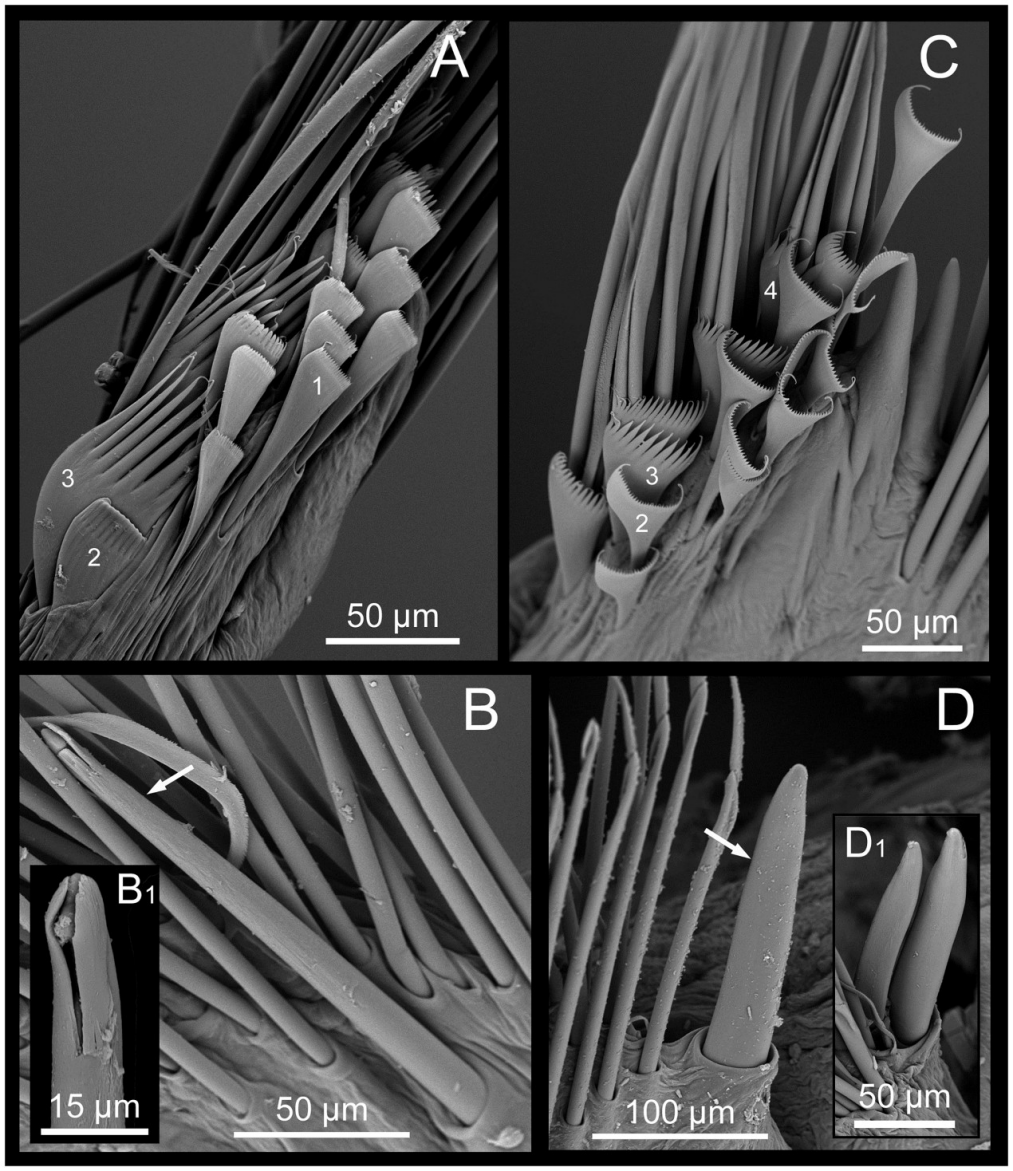
SEM micrographs. *Marphysa gaditana* sp. nov. A. Types of pectinate chaetae from chaetiger 40. B.Bidentate subacicular hook with guards (white arrow). B1. Detail of guards of the bidentate subacicular hook. ***Marphysa chirigota* sp. nov**. C. Types of pectinate chaetae from a posterior–most chaetiger and the acicula with the tips protruding out from acicular lobe. D. Unidentate, subacicular hook lacking guards (white arrow). D1. Detail of a parapodium with two subacicular hooks lacking guards.

**Fig 10:**
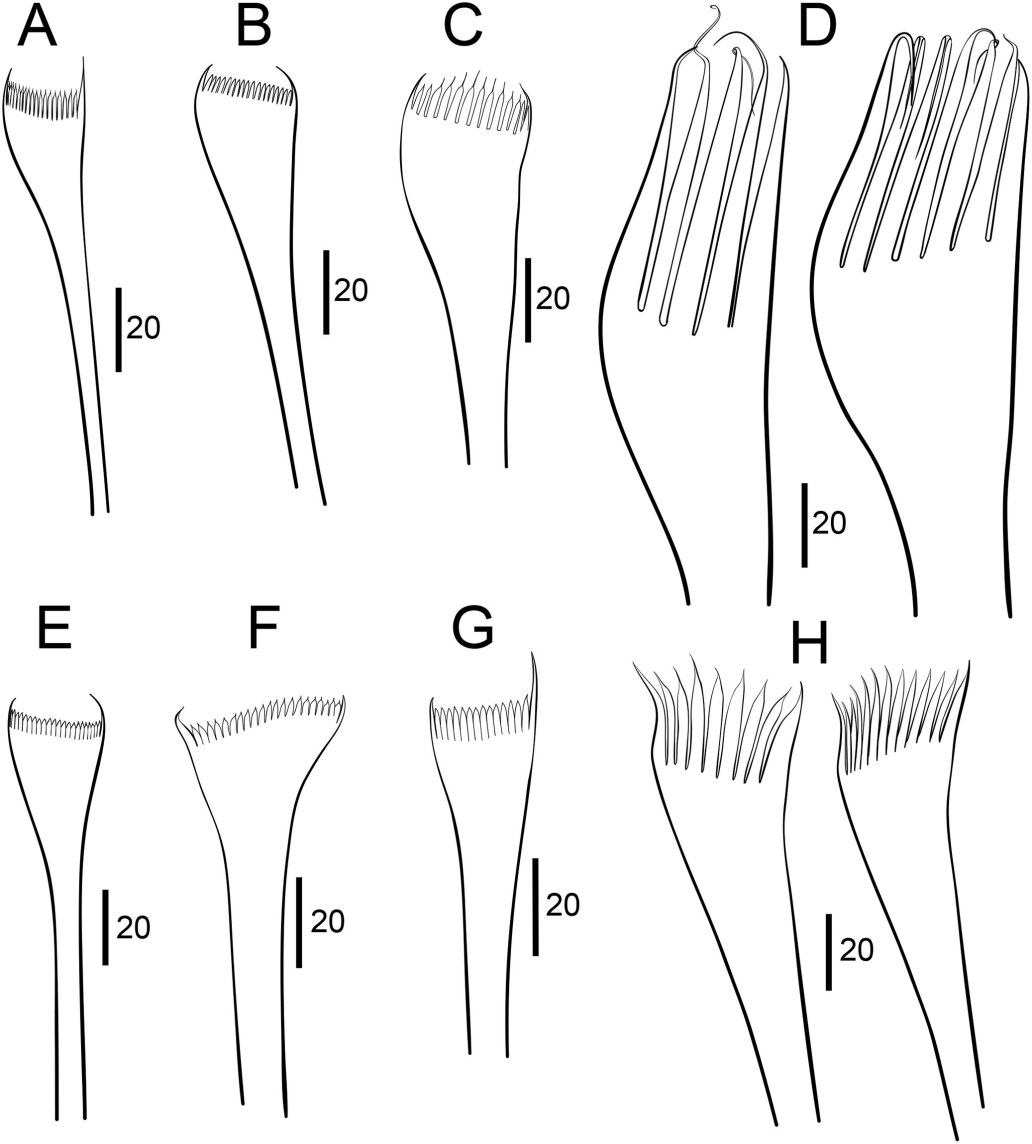
Comparison between pectinate chaetae. A–D. *Marphysa gaditana* sp. nov. E–H. *Marphysa sanguinea* (redrawn from [35]). A,B, E,F: Type 1; C, G: Type 2; D,H: Type 3. Scale bars are μm.

Examined material. Holotype, MNCN 16.01/18522, 4 paratypes, MNCN 16.01/18523 and 1 paratype NHMO C7029; fixed in a 10% buffered seawater formalin solution, preserved in 70% ethanol. The molecular type series contains 2 DNAtypes (MNCN/ADN 118921 and MNCN/ADN 118922), fixed and preserved in 96% ethanol. Same location for all type series: 36° 26’ 32.5”N, 6° 10’ 45.82”W, Salina de San Ramón, Chiclana de la Frontera, Cádiz, SW Iberian Peninsula, 10 cm depth in soft sediment, collected by D. Martin and J. Gil, 10^th^ May 2011. Specimens or their progenitors originally native from intertidal muddy shores at the nearby Natural Park of the Bay of Cádiz (approximate location 36.349°N, 6.181°W).

Description. Holotype long, complete, with ca. 205 chaetigers (ca. 121.7 mm long and 6.5 mm wide at mid–body, without parapodia), slightly widest at median region, abruptly tapering at posterior end (Figs 3A and 4A–4G); round in cross–section until around chaetiger 15, then dorsoventrally flattened. Chaetigers ca. 15 times wider than longer, at widest body region (Figs 3A, 4D and 4E).

Prostomium similar in length to peristomium, narrower than peristomium to as wide as peristomium, about half as deep as peristomium; prostomium dorsoventrally flattened, with anterior end higher and anteriorly bilobate, with a conspicuous median sulcus reaching almost half its length (Figs 3B and 4A–4C). Eyes subdermal, as dots inserted laterally to ceratophores of lateral antennae (Fig 3B), hidden below the anterior peristomial border in preserved specimens (Fig 4A).

Prostomial appendages arranged in semicircle (median and lateral antennae in about the same line, palps a little more anterior), extending beyond prostomium by ca. 2/5 their length (Figs 3A, 3B and 4A–4C). Median antenna, about as long as lateral antennae, all of them directed anteriorly, reaching from middle of chaetiger 2 to posterior border of chaetiger 3 when folded back. Palps about 1/5 shorter than antennae, directed anteriorly reaching from anterior border of chaetiger 2 to middle of chaetiger 3 when folded back. Ceratostyles and palpostyles tapering, lacking peduncle, style basally thicker, non–articulated (Figs 3B and 4A–4C). Ceratophores and palpophores all ring shaped, slightly wider than bases of ceratostyles and palpostyles, almost 12 times shorter than styles length (Fig 3B).

Peristomial rings distinctly separated on all sides; second one about 1/3 and 1/4 of total peristomium length dorsally and ventrally, respectively (Figs 4A–4C). Peristomial ventrolateral lips laterally distinct as elevated surfaces (Figs 4B and 4C). Ventral anterior margin of peristomium forming a shallow arc; lateral margins longer than dorsal side (Fig 4B).

Posterior end of muscularized pharynx reaching chaetigers 8–10. Mandible calcareous cutting plates not seen; sclerotized matrix ca. 10 times shorter than mandible carriers, D–shaped, distally straight, with serrated upper margin and evident growth ring–like marks (Fig 5A). MxI ca. 2.8 times as long as carrier; MxII ca. 3/4 of MxI; MxIII arched, with anterior–most teeth more lateral than posterior–most ones, at least in part ventral to MxII; attachment lamella of MxIII very small, almost not sclerotized; left MxIV wider than longer, triangular; right MxIV longer than wider, arched (Fig 5B). Attachment lamellae of MxIV boomerang–shaped, anterior to plate, left one with arms similar in size and rounded ends, right one with left arm (pointed) more than twice longer than right arm (rounded) (Fig 5B). Maxillary formula: I = 1+1, II = 5+6, III = 5+0, IV = 3+5, V = 1+1. Mx VI absent.

Pre–chaetal lobe shorter than chaetal lobe along whole body. Post–chaetal lobe longer than chaetal lobe in about 40–50 anterior–most chaetigers, conical until chaetiger 5, about 1.5 times longer than wider (Fig 6A and 6B), widely round and about as long as chaetal lobe in most chaetigers, with tapering distal end in posterior–most chaetigers (Fig 4H). Remaining parapodia with post–chaetal lobe shorter than chaetal lobe (Fig 6C–6F).

Notopodial cirri triangular, tapering (almost three times as long as wide at basis), decreasing in length towards posterior end (0.97 mm at chaetiger 5, 0.48 mm at chaetiger 40, 0.25 mm at chaetiger 120), longer than post–chaetal lobes in anterior and median chaetigers shorter than chaetal lobes in posterior chaetigers (Fig 6A–6F) and longer than chaetal lobes in posterior–most chaetigers (Fig 4H). Lateral sense organs as three conspicuously ciliated bumps, located below the notopodial cirri (Fig 7A). Ventral cirri thumb–shaped with round wide tips; with inflated bases all along body from chaetiger 6 except about last 20, being round to triangular, with distinct round tips (Fig 6A–6F). Ventral cirri about 2/3 as long as notopodial cirri in anterior–most chaetigers, decreasing in length towards posterior end (0.58 mm at chaetiger 5, 0.55 mm at chaetiger 40, 0.35 mm at chaetiger 120).

Branchiae palmate (Fig 6C–6F), starting around chaetiger 20–25, with one filament in the first 1(2) branchial chaetigers, 2–3 in the initial 17% of body, then a maximum of 4–5 from chaetiger 39–160, three from chaetiger 161–170, and 1–2 from chaetiger 171–195; last branchiae on posterior 15% of body, ca. 35 chaetigers before pygidium. Best–developed branchiae with longest branchial filament around 7.5 times longer than notopodial cirri and 2.7 and 6 times longer than branchial stem length and branchiae basal width, respectively.

Notopodial aciculae in all notopodial cirri from second body quarter, pale brown, almost inconspicuous. Neurochaetal lobe round all along body, with a more or less marked middle incision giving a bilobed appearance. Chaetae distributed in two distinct bundles: supracicular, with limbate and pectinate chaetae at anterior edge, and subacicular, with compound spiniger chaetae and subacicular hooks (Fig 7C). Neuroaciculae blunt to tapering, golden brown, placed dorsal to midline in anterior-most parapodia and on midline thereafter; distributed in an oblique row, with anterior–most neuroacicula being also dorsal–most in parapodia. 1–2 neuroaciculae per parapodium in parapodia 1, 3(2) from parapodia 2 to 5, 4(3) to parapodia 15, 3 to parapodia 40, 1–2 in median and posterior regions. Number of limbate and compound spinigers decreasing towards posterior end. Limbate chaetae with proximal end and flat margin of distal end finely serrated (Fig 8A); anterior–most limbate chaetae in bundle shortest. Compound spiniger chaetae with finely serrated shafts and blades; blades flat, varying in length within bundle (Fig 8B and 8C). Compound spinigers inserted at anterior–most row of bundle, dorsal–most one slightly more dorsal than ventral–most neuroacicula. Pectinate chaetae in all chaetigers (Figs 7C, 8G–8I, 9A and 10A–10D), inserted between dorsal bundle of limbates and neuroaciculae, with a similar position all along the body; pectinate chaetae of three types: 1) 8–10 thin, flat to little curved, lightly serrated, isodont with external teeth slightly differing in length, slightly asymmetrical (almost symmetrical in anterior–most chaetigers), with ca. 17–22 teeth, varying in length on different chaetae, evenly tapering (Type 1, Figs 8G, 9A, 10A and 10B); 2) 4–6 thick, flat to little curved, isodont, slightly asymmetrical, with 10–14 teeth, coarse and long, with short filiform tips and variable lengths on different chaetae (Type 2, Figs 8H, 9A and 10C); 3) 3–6 thick, very large, non–curved chaetae, anodont, asymmetrical, resembling a hair pick, with 5–10 teeth, very long, coarse, tapering to very long filiform ends, ca. ten times longer than wider (Type 3, Figs 8I, 9A and 10D), absent from anterior most chaetigers. Pseudo–compound chaetae absent. Subacicular hooks first present from chaetigers 40–55, absent from some parapodia, usually one per parapodium, dark yellow, bidentate, with round tips and two guards covering tip, ca. twice thicker than shaft of spinigers (Figs 7C, 8D, 8F, 9B and 9B1); when present, second hook unidentate, guards absent (Fig 8E).

Pygidium longer on ventral side, with two pairs of tapering pygidial cirri on ventral side; dorsal pygidial cirri ca. 5–10 times longer than ventral ones (Fig 4F–4H).

Remarks. In addition to the marked molecular differences found in our analyses (Figs 1 and 2) and the distinct biogeographical origin [8], *M*. *gaditana* sp. nov. is characterised by having bidentate subacicular hooks. Thus it can be clearly distinguished from the species of the *sanguinea*–group having them i) unidentate (see a full list in the remarks on *M*. *chirigota* sp. nov.), ii) unidentate to faintly bidentate (*M*. *kristiani* Zanol, da Silva & Hutchings, 2016 [52]), iii) bidentate but present only in the last parapodia (*M*. *hongkongensa* Wang, Zhang & Qiu, 2018 [51]), or iv) absent, at least in large adults (*M*. *californica* Moore, 1909 [47], M. brevitentaculata Treadwell, 1921 [45, 81], *M*. *victori* Lavesque, Daffe, Bonifácio & Hutchings, 2017 [3]). It can also be distinguished from *M*. *multipectinata* Liu, Hutchings & Sun, 2017 [34], which has subacicular hooks starting at chaetiger 20 (vs. 40–55 in our new species). *Marphysa tribranchiata* Liu, Hutchings & Sun, 2017 [34] and *M*. *schmardai* Gravier, 1907 [82] have a maximum of three branchial filaments (vs. 4–5 in our new species). *Marphysa brasiliensis* (Hansen, 1882) [83] and *M*. *mullawa* Hutchings & Karageorgopolous 2003 [29] have branchiae starting from chaetiger 28–33 and *M*. *acicularum* Webster, 1884 [84] from chaetigers 27–35 (vs. 40–55 in *M*. *gaditana* sp. nov.). *Marphysa viridis* Treadwell, 1917 [56] has one type of isodont pectinate chaetae (vs. two in *M*. *gaditana* sp. nov.), being less numerous (4–5 vs. 8–10) and showing a lower number of teeth (14 vs. 22) in middle and posterior regions. Our new species can also be distinguished from *M*. *elityeni* Lewis & Karageorgopoulos, 2008 [30], whose subacicular hooks start after chaetiger 60 (instead of before).

The five following species, normally included or associated with the *sanguinea*–group and mainly described from European waters, have been discarded due to incomplete original descriptions and lack of redescriptions, which prevents a comparison: *Leodice opalina* Savigny *in* Lamarck, 1818 [85] (probably from Atlantic coast of France), *Leodice erithrocephala* Risso, 1826 [86] (Nice region, Mediterranean coast of France), *Leodice grunwaldi* Risso, 1826 [86] (Nice, Mediterranean coast of France), *Lysidice multicirrata* Claparède, 1863 [87] (St. Vaast la Hougue, Atlantic coast of France), and *Marphysa haemasona* Quatrefages, 1866 [19] (South Africa).

Morphologically, *M*. *gaditana* sp. nov. most closely resembles the recently redescribed *M*. *sanguinea* [29, 35, 36], but differs, among other characters, in having some parapodia with two subacicular hooks, the second one being unidentate (instead of only one, bidentate in *M*. *sanguinea*), in having three types of pectinate chaetae in posterior parapodia (instead of only two, with Type 1 lacking, in *M*. *sanguinea*) and in the shape of the anodont pectinate chaetae from posterior chaetigers, which are very large and have 5–10 teeth with filiform tips (instead of normal size, 6–14 teeth, lacking filiform tips in *M*. *sanguinea*) (Fig 10A–10H).

Despite the absence of reliable evidences, it has been suggested that the presence of a secondary subacicular hook in some parapodia in the species of *Marphysa* could represent a replacement for the main one [81]. However, the fact that, when present, the secondary hook in *M*. *gaditana* sp. nov. is unidentate and lacks guards, while the main one is bidentate and has a pair of guards, casts some doubts on this replacement hypothesis. The presence of subacicular hooks seems to be a variable character within a given specimen, as they may also be absent from some parapodia (after first appearing). Therefore, we strongly recommend to consider this variability as a relevant character in species description.

*Marphysa gaditana* sp. nov. differs from *M*. *chirigota* sp. nov. and *M*. *aegypti* in having bidentate subacicular hooks with guards (unidentate in the other two species). All molecular species delimitation methods used herein grouped the COI sequences of *M*. *gaditana* sp. nov. with those in GenBank from Cap de la Hague (France), Sado Estuary (Portugal), and Florida and Virginia (USA), all them in Atlantic waters. It is feasible that the first two localities fall within the natural species distribution area (particularly the second one), but the records from the USA are certainly surprising. This wide disjoint distribution is uncommon for the family and deserves further investigation.

Etymology. The specific epithet refers to *Gadir*, the Fenician name of the oldest settlement of the city of Càdiz; “*gaditana*” means “from Gadir” and it is the Spanish epithet (feminine) for Cádiz inhabitants.

Distribution. Type materials collected at the Salina de San Ramón; however, according to ICZN Article 76.1.1 [88], the type locality must be the nearby intertidal muddy shores of the Natural Park of the Bay of Cádiz (approx. 36.349°N, 6.181°W), Chiclana de la Frontera, Cádiz (SW Iberian Peninsula), from where the specimens or their progenitors were originally native. Localities of samples identified as the same species based on molecular evidence, all them in the Atlantic Ocean: Cap de la Hague (France), Sado Estuary (Portugal), Florida and Virginia (USA).

#### *Marphysa chirigota* Martin, Gil and Zanol sp. nov.

LSID: urn:lsid:zoobank.org:act:90486B6A-CB92-4284-A97C-7B2381DAF4D0 Figs 3C, 3D, 5C, 5D, 7B–7D, 9C, 9D, 11–13 and 14A–14D.

**Fig 11:**
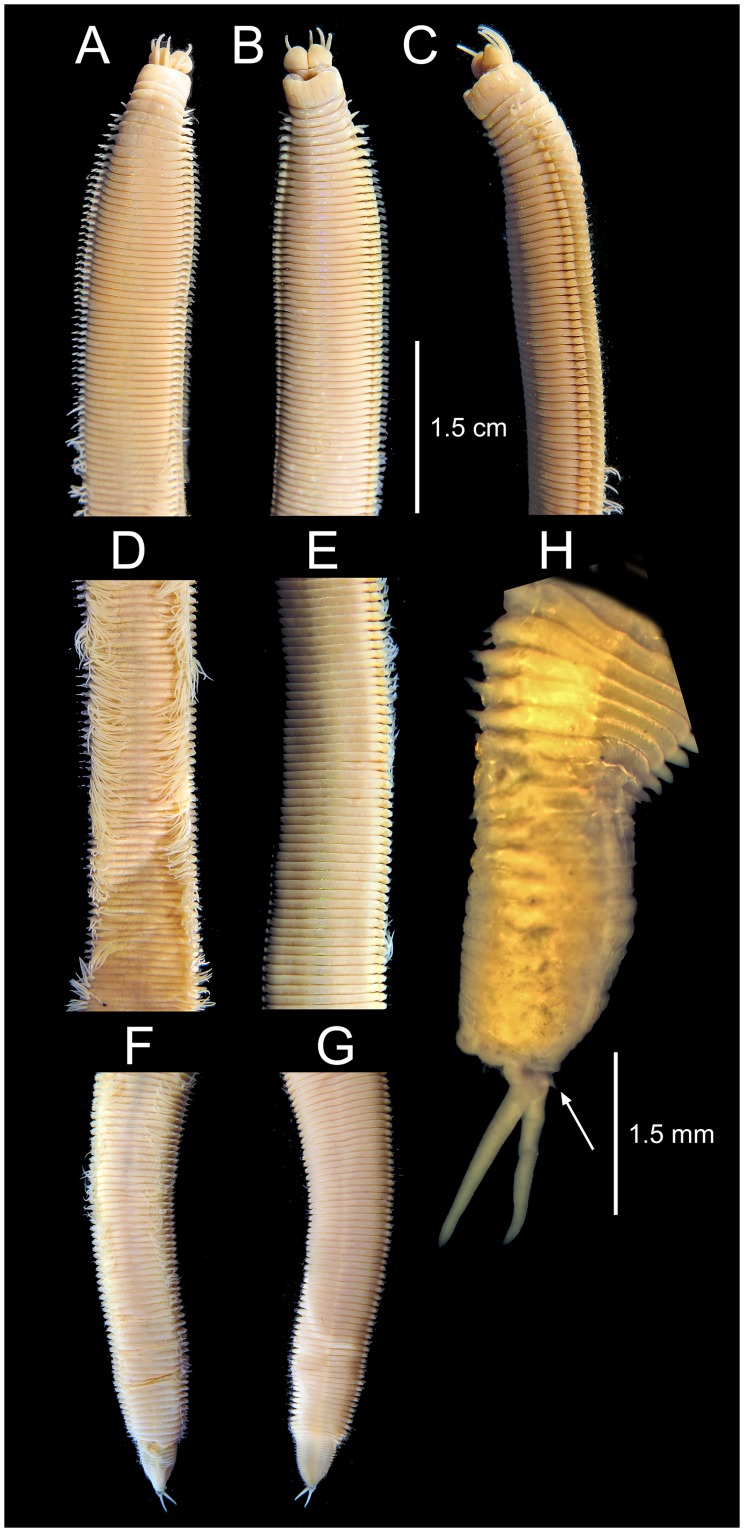
*Marphysa chirigota* sp. nov. Anterior end. A. Dorsal view. B. Ventral view. C. Lateral view. Mid–body. D. Dorsal view. E. Ventral view. Posterior end. F. Lateral view. G. Ventral view. H. Detail of pygidium showing the two pairs of anal cirri. A–G same scale.

**Fig 12:**
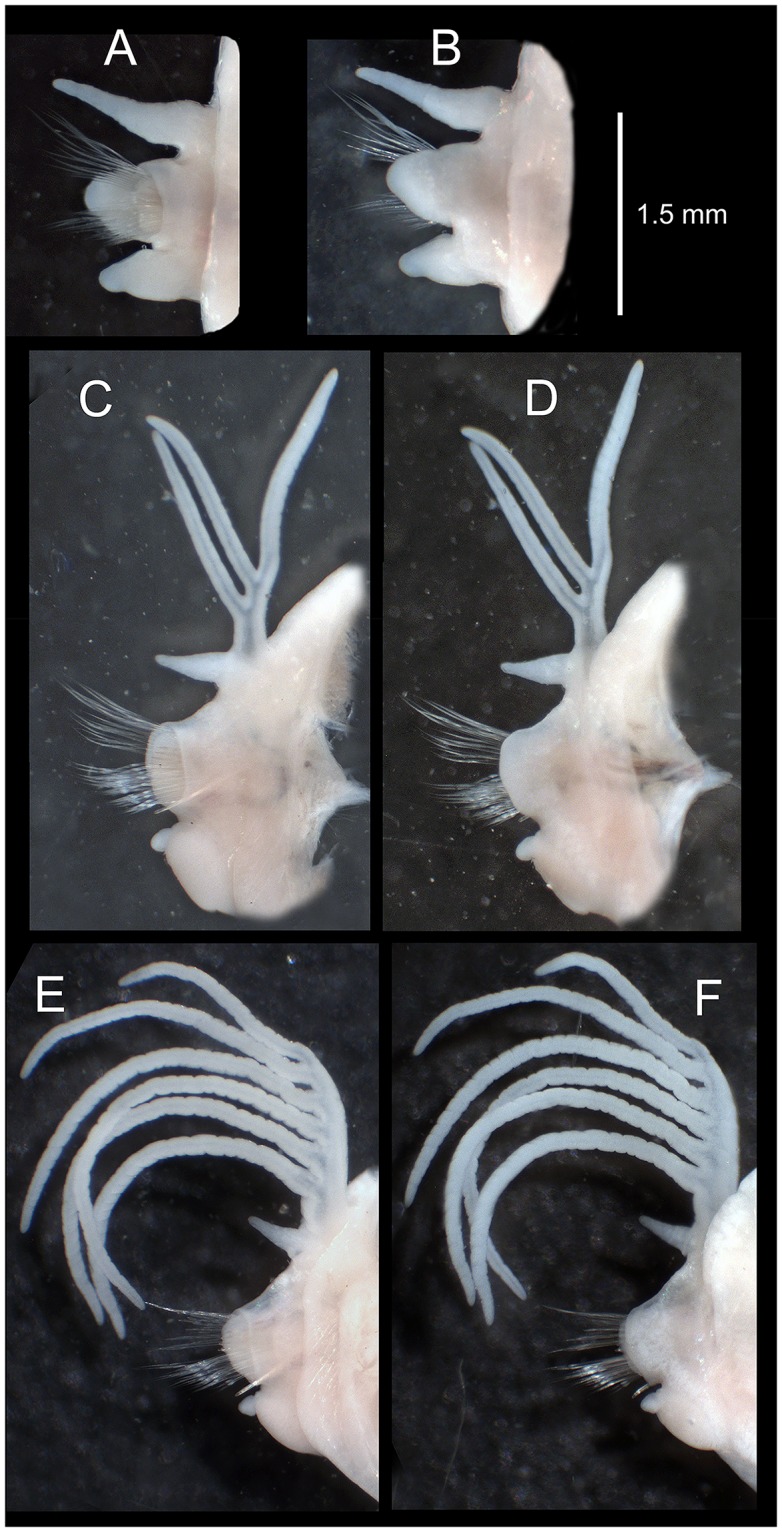
*Marphysa chirigota* sp. nov. Parapodium from chaetiger 5 in antero–posterior (A) and postero–anterior (B) views. Parapodium from chaetiger 40 in antero–posterior (C) and postero–anterior (D) views. Parapodium from a posterior chaetiger (130) in antero–posterior (E) and postero–anterior (F) views.

**Fig 13:**
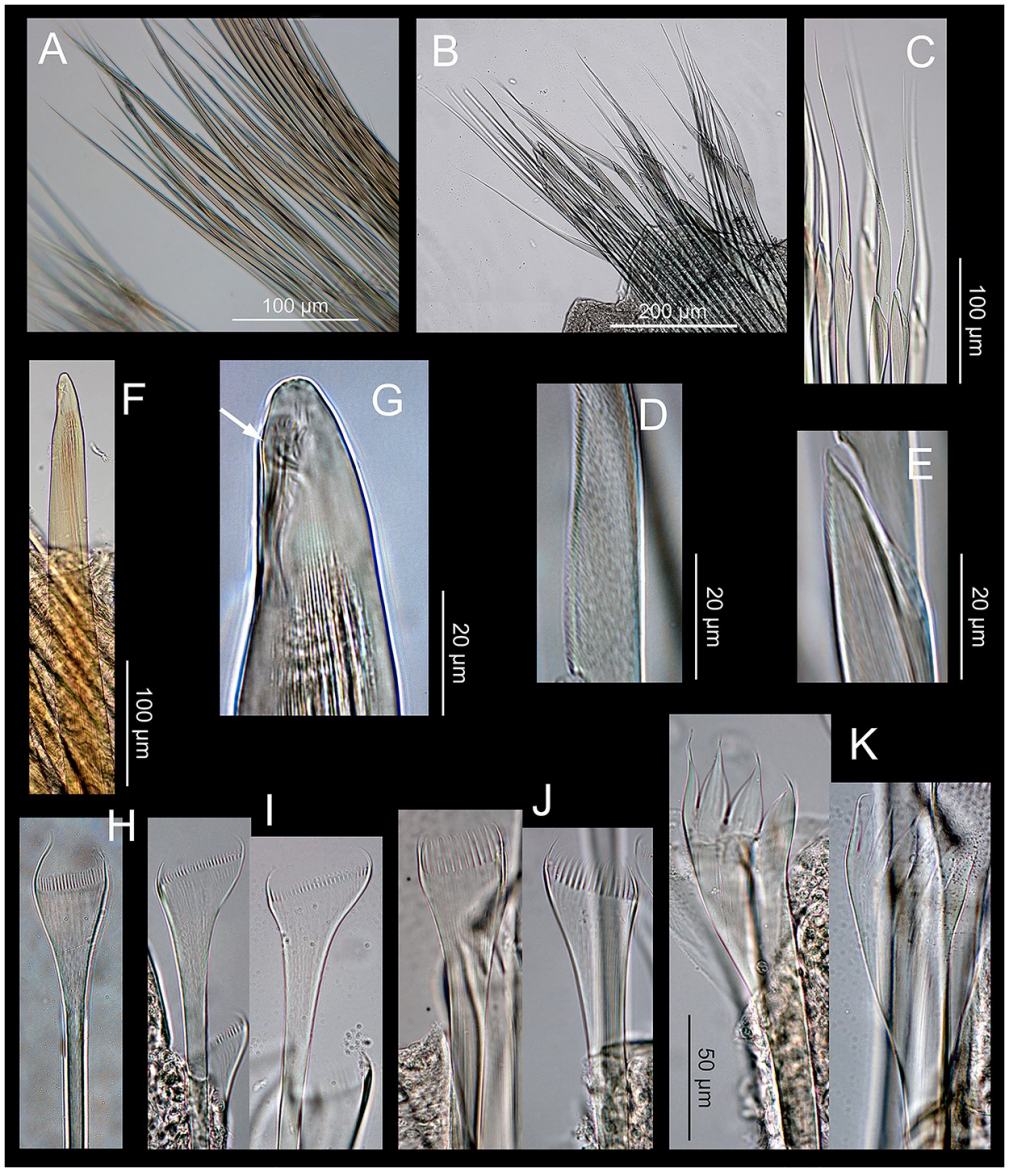
*Marphysa chirigota* sp. nov. A. Supracicular limbate chaetae. B. Subacicular spiniger compound chaetae. C. Close view of spiniger compound chaetae. D. Detail of blade serration of spiniger compound chaeta. E. Detail of shaft tip serration of spiniger compound chaeta. F. Unidentate subacicular hook. G. Detail of the tip of the unidentate subacicular hook; white arrow pointing on guards. H. Type 1 pectinate chaeta. I. Type 2 pectinate chaetae. J. Type 3 pectinate chaetae; K. Type 4 pectinate chaetae.

**Fig 14:**
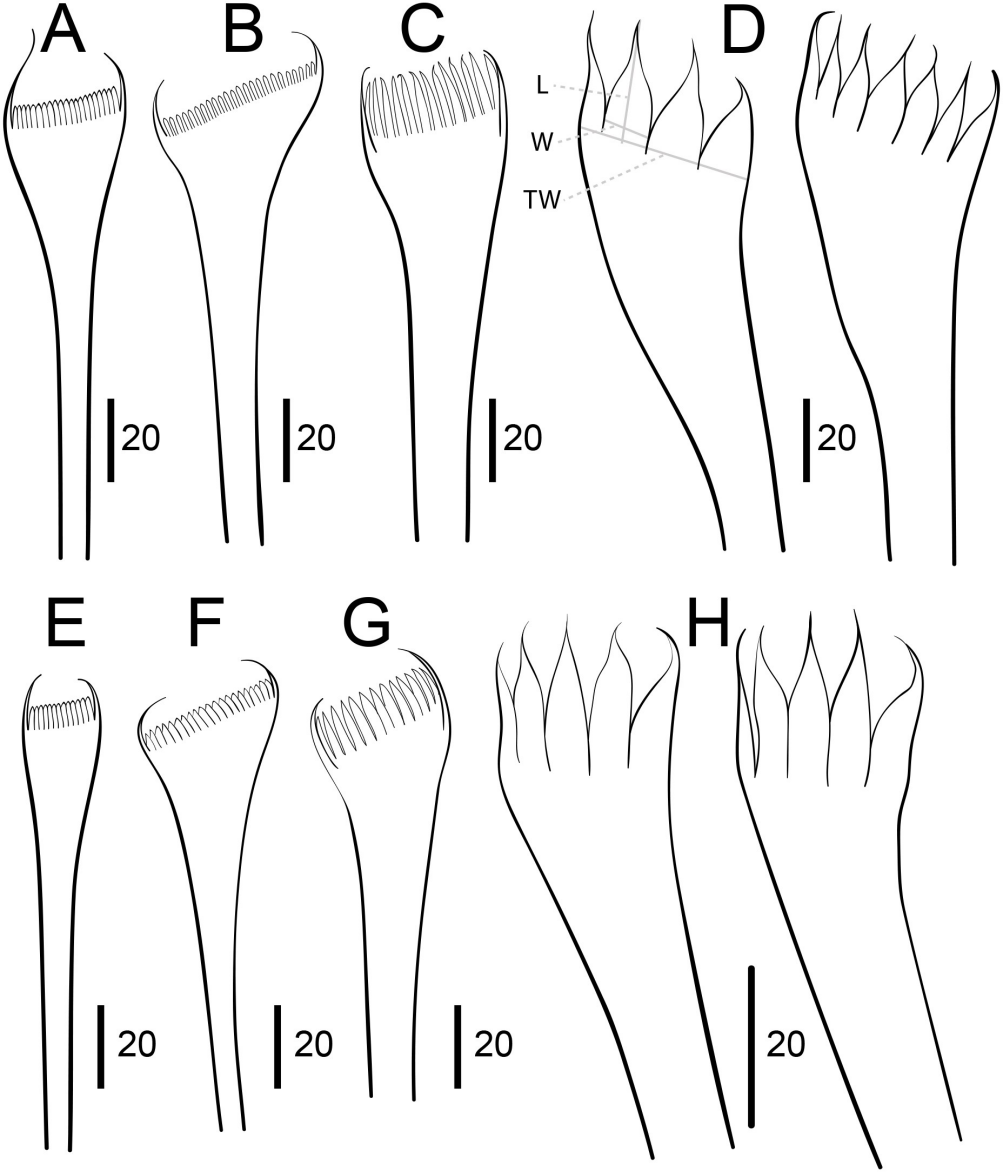
Comparison between pectinate chaetae. A–D. *Marphysa chirigota* sp. nov. E-H *Marphysa aegypti* (redrawn from [31]); A, E: Type 1; B, F: Type 2; C, G: Type 3; D, H: Type 4. L: length of teeth; W: width of teeth; TW: width of chaetal tip. Scale bars are μm.

Examined material. Holotype, MNCN 16.01/18524, 4 Paratypes, MNCN 16.01/18525, and 1 paratype, NHMO C7030; fixed in a 10% buffered seawater formalin solution, preserved in 70% ethanol. The molecular type series contains 3 DNAtypes (MNCN/ADN 118918 to MNCN/ADN 118920), fixed and preserved in 96% ethanol. Same location for all type series:36° 26’ 32.5”N, 6° 10’ 45.82”W, Salina de San Ramón, Chiclana de la Frontera, Cádiz, SW Iberian Peninsula, 10 cm depth in soft sediment, collected by D. Martin and J. Gil. Specimens or their progenitors originally native from intertidal sandy shores of the nearby Natural Park of the Bay of Cádiz (approx. 36.349°N, 6.181°W).

Description. Holotype complete, very long, with ca. 370 chaetigers (26.5 cm long, 7.9 mm wide at mid–body, without parapodia), slightly widest all along median region, progressively tapering at regenerating posterior end (Figs 3C and 11A–11G); round in cross–section until around chaetiger 20–25, then dorsoventrally flattened (Fig 3C). Chaetigers more than 13 times wider than longer at widest body region (Figs 11D and 11E).

Prostomium ca. 1/3 shorter than, and as wide as, peristomium, about half as deep as peristomium. Prostomium dorsoventrally flattened, with anterior end higher and anteriorly bilobate, with a conspicuous median sulcus reaching almost 1/3 its length (Figs 3D and 11A). Eyes subdermal, as dots inserted laterally to lateral antennae ceratophores (Fig 3D), hidden below the anterior peristomial border in preserved specimens (Figs 3D and 11A).

Prostomial appendages arranged in semicircle (median and lateral antennae in the same line, palps a little more anterior), extending beyond prostomium between half and 2/3 their length (Figs 3D and 11A). Median antenna, about as long as lateral antennae, all of them directed anteriorly, reaching from middle of chaetiger 1 to posterior border of chaetiger 3 when folded back. Palps about 1/5 shorter than antennae, directed anteriorly, reaching from middle of chaetiger 1 to middle of chaetiger 3 when folded back. Ceratostyles and palpostyles tapering, lacking peduncle, style basally thicker, non–articulated (Figs 3D and 11A). Ceratophores and palpophores all ring shaped, slightly wider than bases of ceratostyles and palpostyles, almost 13 times shorter than styles length (Fig 3D).

Peristomial rings distinctly separated on all sides; second one about 1/3 of total peristomium length (Figs 3D and 11A–11C). Peristomial ventrolateral lips laterally distinct as elevated surfaces (Figs 11B and 11C). Ventral anterior margin of peristomium forming a shallow arc; lateral margins longer than dorsal side (Figs 3D and 11A–11C).

Posterior end of muscularized pharynx reaching chaetigers 5–6. Mandible calcareous cutting plates not seen; sclerotized matrix ca. 13 times shorter than mandible carriers, D–shaped, distally straight, with serrated upper margin and evident growth ring–like marks (Fig 5C). MxI ca. 2.5 times as long as carrier; MxII ca. 3/4 of MxI; MxIII arched, with anterior–most teeth more lateral than posterior–most ones, ventral to MxII; attachment lamella of MxIII short, as an elongated D, strongly sclerotized, placed at middle of plate ventral edge; MxIV longer than wider, left one short, straight; right one, arched; attachment lamellae of MxIV roughly C–shaped, ventral to plate, left one with left arm (pointed) more than twice shorter than right arm (rounded), right one with left arm (pointed) ca. four times longer than right (rounded) (Fig 5D). Maxillary formula: I = 1+1, II = 4/5+5, III = 6+0, IV = 4/5+7, V = 1+1. Mx VI absent.

Pre–chaetal lobe shorter than chaetal one along whole body. Post–chaetal lobe longer than chaetal one in about 40–50 anterior–most chaetigers, finger–like until chaetiger 4, blunt triangular on chaetiger 5, about 1.5 wider than longer (Fig 12A and 12B), wide rounded and about as long as chaetal lobe in most chaetigers (Fig 12C–12F), with tapering distal end in posterior–most chaetigers.

Notopodial cirri triangular, tapering (almost three times longer than wide at basis), decreasing in length towards posterior end (0.97 mm at chaetiger 5, 0.48 mm at chaetiger 40, 0.25 mm at chaetiger 120), longer than post–chaetal lobes in anterior chaetigers, as long as in median chaetigers and shorter in posterior ones (Fig 12A–12F), but longer than post–chaetal lobes in posterior–most chaetigers (Figs 11F and 11G). Lateral sense organs as single ciliated bump, located below the notopodial cirri (Fig 7B). Ventral cirri thumb–shaped with round wide tips; from chaetiger 5 with inflated bases along most of the body, except about last 20, being round to triangular, with distinct round tips (Fig 12A–12F). Ventral cirri about half as long as notopodial cirri in anterior–most chaetigers (0.59 mm at chaetiger 5), similar in length in maximum branchial development region (0.54 at chaetiger 40) and decreasing in length at end of branchial region (0.43 mm at chaetiger 120).

Branchiae palmate (Fig 12C–12F), starting around chaetiger 25–30, with one filament in the first 1(2) branchial chaetigers, 3–4 up to the initial 17% of body, five from chaetiger ca. 55 to 75, a maximum of six until ca. 220, then 3–4 until ca. 280 and 1–2 until ca. 330; last branchiae on posterior 10% of body, ca. 40 chaetigers before pygidium. Best–developed branchiae with longest branchial filament around 8 times longer than notopodial cirri and 2.7 and 10 times longer than branchial stem length and branchiae basal width, respectively.

Notopodial aciculae in all notopodial cirri along second body quarter, pale yellow, inconspicuous. Neurochaetal lobe round all along body. Chaetae distributed in two distinct bundles: supracicular with limbate and pectinate chaetae at anterior edge, and subacicular with compound spiniger chaetae and subacicular hooks (Fig 7D). Neuroaciculae blunt to tapering, dorsal to parapodia midline in anterior segments and along parapodia midline thereafter; distributed in an oblique row, with anterior–most neuroacicula being also dorsal–most in parapodia, with tips clearly protruding from acicular lobe (Fig 9C). Three neuroaciculae per parapodium on chaetiger 1, 3–4 until chaetiger 30, then 4–6 to parapodia 120, 3–4 to ca. chaetiger 320, then 3(2) to body end. Neuroaciculae golden brown (Fig 7D). Number of limbate chaetae and compound spinigers decreasing towards posterior end. Limbate chaetae with proximal end and flat margin of distal end serrated; anterior–most limbate chaetae in bundle shortest (Fig 13A). Compound spiniger chaetae with serrated shafts and blades; blades flat, varying in length within bundle (Fig 13B–13E). Anterior parapodia with dorsal–most compound spiniger chaetae inserted at anterior–most row of bundle, as dorsal as ventral–most neuroacicula. Pectinate chaetae in all chaetigers except in first four, inserted between dorsal bundle of limbate chaetae and neuroaciculae (Figs 7D and 9C); pectinate chaetae of four types; i) 2–10 thin, flat to little curved, lightly serrated chaetae, with evenly tapering fine teeth, isodont with external teeth markedly differing in length, with ca. 20–30 teeth, number of chaetae and teeth increasing towards midbody, (Type 1, Figs 13H and 14A); ii) 2–10 thin, flat to little curved, lightly serrated chaetae, isodont, with ca. 20–30 evenly tapering fine teeth, number of chaetae and teeth and degree of asymmetry increasing from anterior to posterior parapodia (Type 2, Figs 13I and 14B); iii) 5–6 thick, flat to little curved chaetae, markedly asymmetrical, isodont, with 13–16 coarse and long teeth, of variable length on different chaetae (Type 3, Figs 13J and 14C); iv) 2–5 thick, large, non–curved, asymmetrical chaetae resembling a hair pick, anodont, with 4–7 thick, almost triangular teeth, tapering to filiform ends, 3–5 times longer than wider (Type 4, Figs 13K and 14D). Type 1 present on anterior–most parapodia, being progressively replaced by Type 2, present alone on roughly half anterior body; Types 3 and 4 appearing around mid–body and on posterior–most parapodia, respectively; Type 4 with teeth length *vs*. tip width ratio of 0.5–8.0 and teeth length *vs*. width ratio of 2.5 (Figs 13K and 14D). Pseudocompound chaetae absent. Subacicular hooks ca. four times thicker than shaft of spinigers, first present from chaetiger 30–45, then in all posterior chaetigers, usually one per parapodium, two in some posterior–most chaetigers, dark yellow, unidentate, with round tip, one with guards absent, another with guards absent or very small (Figs 7D, 9D, 9D1, 13F and 13G).

Pygidium longer on ventral side, with two pairs of tapering pygidial cirri on ventral side; dorsal pygidial cirri ca.14 times longer than ventral ones (Fig 11H).

Remarks. In addition to the marked molecular differences found in our analyses (Figs 1 and 2) and the distinct biogeographical origin [8], *M*. *chirigota* sp. nov. differs from all species of the *sanguinea*–group either having bidentate subacicular hooks with guards, or laking them at all (see remarks on *M*. *gaditana*). It also differs from *M*. *bulla* Liu, Hutchings & Kupriyanova, 2018 [33], *M*. *nobilis* Treadwell, 1917 [56] and *M*. *tripectinata* Liu, Hutchings & Sun, 2017 [34] in having subacicular hooks starting at chaetiger 30–45 vs. 71, 255 and 170, respectively. *Marphysa aransensis* Treadwell, 1939 [89] has less isodont pectinate chaetae in anterior segments (1–2 vs. 2–10), isodont and anodont pectinate chaetae in middle parapodia (instead of two isodont types) and less numerous pectinate chaetae in posterior parapodia, where the anodont ones have 14 teeth (vs. 4–7 in *M*. *chirigota* sp. nov.). *Marphysa furcellata* Crossland, 1903 [22], *M*. *iloiloensis* Glasby, Mandario, Burghardt, Kupriyanova, Gunton & Hutchings, 2019 [8], and *M*. *mangeri* Augener, 1918 [90] have the first branchial segments before chaetiger 25 and *M*. *macintoshi* Crossland, 1903 [22] and *M*. *tamurai* Okuda, 1934 [27] after chaetiger 30 (25–30 in *M*. *chirigota* sp. nov.). *Marphysa parishii* Baird, 1869 [91] was described as having pectinate chaetae appearing only in the posterior body region (vs. from first chaetigers in *M*. *chirigota* sp. nov.) and *Marphysa acicularum brevibranchiata* Treadwell, 1921 [45] has 6+6 and 8+9 teeth in the maxilla II and IV (vs. 4/5+5 and 5/5+7 in *M*. *chirigota* sp. nov.).

In turn, *Leodice opalina* Savigny *in* Lamarck, 1818 [85], *Leodice erithrocephala* Risso, 1826 [86], *Leodice grunwaldi* Risso, 1826 [86], *Lysidice multicirrata* Claparède, 1863 [87] and *Marphysa haemasona* Quatrefages, 1866 [19] are discarded for the same reasons discussed in the remarks for *M*. *gaditana* sp. nov.

*Marphysa chirigota* sp. nov. most closely resembles the recently described *M*. *aegypti* in overall body size and in having very robust, unidentate subacicular hooks (single in most parapodia, two—both similar in size and form—in some posterior parapodia). However both species differ in numerous morphological characters: the ratio chaetiger width/length, the ratio prostomium/peristomium length, the presence of peduncle in cerato–and palpostyles, the posterior end of muscularised pharynx, the maxillary formula, the shape of notopodial cirri, the length and proportions of branchial filaments, the number and colour of neuropodial acicula, and the shape and number of pectinate chaetae (Table 2, Fig 14). Despite the numerous differences, distinguishing the two species requires a careful observation of key characters. These subtle differences are also reflected in the low genetic differentiation between both species (2.9–3.74%), which are borderline between intraspecific and interspecific for polychaete species [92, 93], suggesting a recent speciation event.

**Table 2:** Summary of the main differences between *M*. *aegypti* and *M*. *chirigota* sp. nov. based on descriptions and observation of type material.

	*M*. *aegypti*	*M*. *chirigota* sp. nov.
Chaetiger number	293	370
Body length	143 mm	265 mm
Body width	9 mm	7.9 mm
Chaetigers width *vs*. length	7 times	13 times
Length prostomium *vs*. peristomium	Equal	1/3
Styles	digitiform, with peduncle	tapering, lacking peduncle
Posterior end of muscularized pharynx	up to chaetiger 4	up to chaetiger 6
Mx I	1+1; dark, with white tips	1+1; dark brown
Mx II	4+4	4/5+5
Mx III	5+0	6+0
Mx IV	4+6	4/5+7
Mx V	2+1	1+1
Notopodial cirri	digitiform; longer than chaetal lobes along whole body	triangular; longer (anterior), as long as (median), shorter (posterior) and longer (posterior-most) than chaetal lobes
Branchial filaments	3 times longer than notopodial cirri	8 times longer than notopodial cirri
	2 times longer than branchial stems	2.7 times longer than branchial stems
Neuropodial acicular	3 in all parapodia, black	up to 6, golden brown
Pectinate chaetae		
Type 1		
Shape	isodont (with external teeth markedly differing in length), symmetrical	isodont (with external teeth markedly differing in length), symmetrical
Number of teeth	≈15	≈25
Type 2		
Shape	isodont, asymmetrical	isodont, asymmetrical
Number of teeth	< 25	> 25
Tip width	35 μm	45 μm
Teeth length *vs*. tip width	0.18	0.10
Type 3		
Shape	isodont, asymmetrical	isodont, asymmetrical
Teeth tips	pointed	slightly filiform
Type 4		
Shape	anodont, asymmetrical	anodont, asymmetrical
Number of chaetae	2	4–5
Number of teeth	5	4–7
Tip width	< 25 μm	> 45 μm
Teeth length *vs*. tip width	1	0.5–0.8
Teeth length *vs*. width	4	2.5

Etymology. The specific epithet “chirigota” is a tribute to the “Chirigotas”, a genre of choral folksong typical of Cádiz province (SW Iberian Peninsula), the type locality of the species. The chirigotas are satirical-humoristic songs performed predominantly in the streets by costumed performers during Carnival, and reflect much of the refined local sense of humour and hospitality that the first two authors had the chance to enjoy during the collection trip. By selecting this species name, all authors aim to contribute promoting the amazing heritage (natural, cultural and human) of the whole region of the Gulf of Cádiz.

Distribution. Type material collected at the Salina de San Ramón; according to ICZN Article 76.1.1 [88], the type locality must be considered as the nearby intertidal sandy shores of the Natural Park of the Bay of Cádiz (approx. 36.349°N, 6.181°W), Chiclana de la Frontera, Cádiz (Iberian Peninsula), from), where the specimens or their progenitors were originally native. The species is also likely to be present in south Portugal, namely in Parchal (Algarve). This area is characterised by intertidal sand banks in marine sheltered waters (similar to the type locality) and harbours a population of *Marphysa* with very long specimens, known by Portuguese anglers as “ganso do Parchal” (Nuno Lopes, pers. comm. 28 April 2019).

#### *Marphysa aegypti* Elgetany, El-Ghobashy, Ghoneim and Struck, 2018 [31]

**Fig 15:**
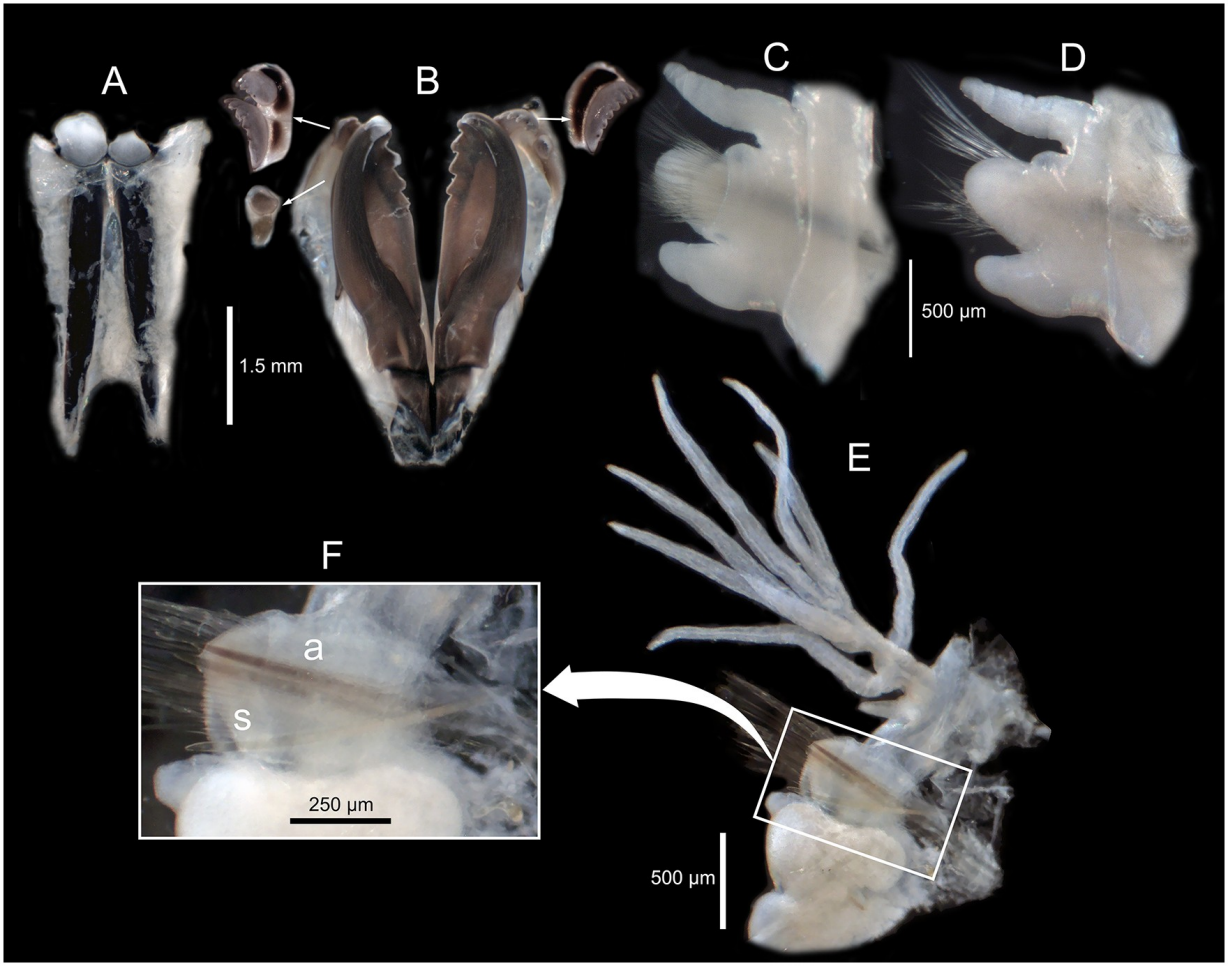
*Marphysa aegypti*. Paratype NHMO C6963. A. Dissected mandible. B. Dissected maxillae. **Paratype NHMO C6965**: C. Anterior parapodium (chaetiger 5), anterior view. D. Same parapodium, posterior view. **Paratype NHMO C6964**: E. Mid–posterior parapodium (chaetiger 100), anterior view; F. Detail of same parapodium as E, showing the position of the neuroacicula (a) and the subacicular hook (s).

**Fig 16:**
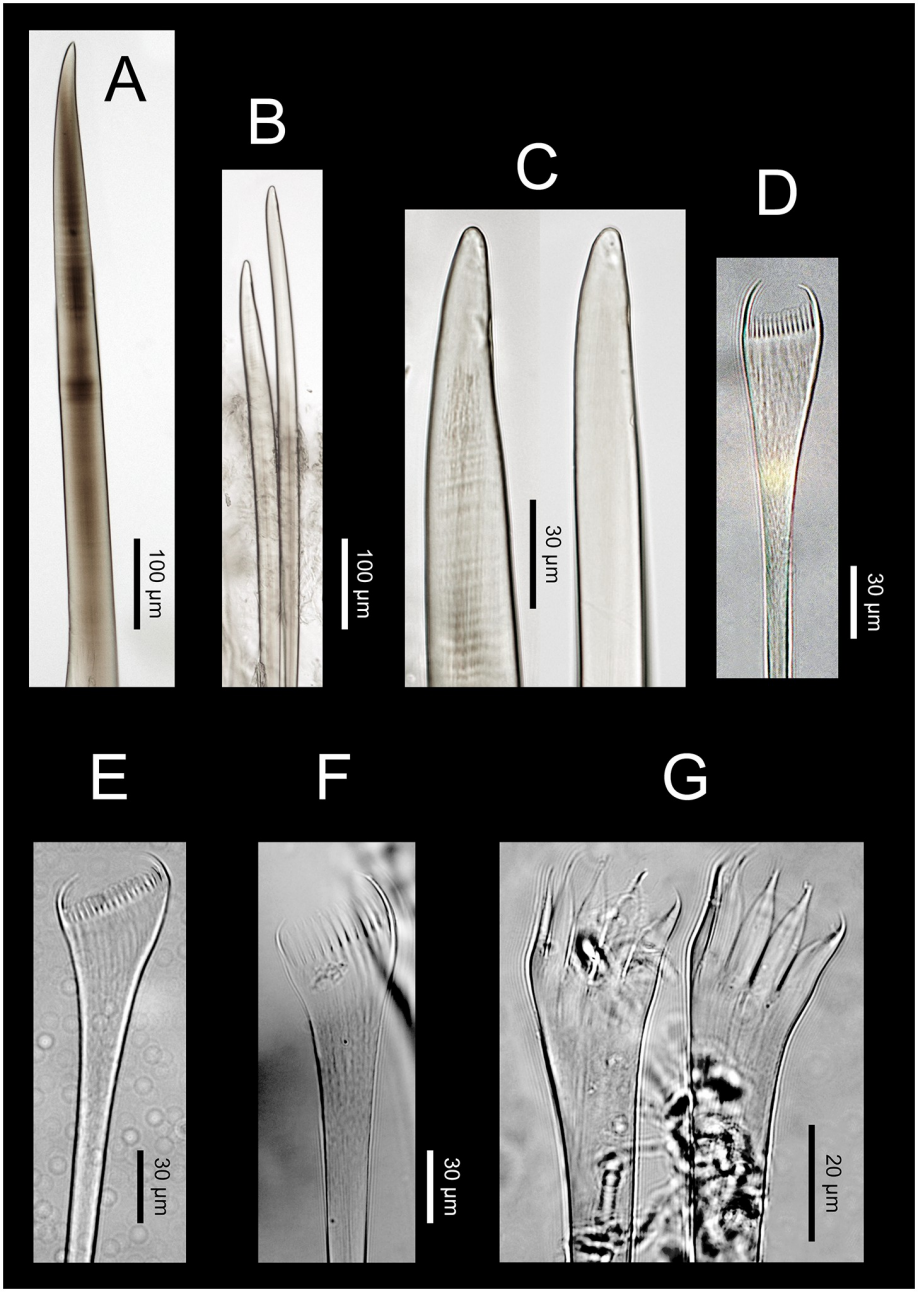
*Marphysa aegypti*. Paratype NHMO C6964. A. Neuroaciculum. B. Two unidentate subacicular hooks from same parapodium. C. Detail of tips of subacicular hooks in B. D. Type 1 pectinate chaeta. E. Type 2 pectinate chaeta. F. Type 3 pectinate chaetae; G. Type 4 pectinate chaetae.

LISID: urn:lsid:zoobank.org:act:EC8C5797-11DB-45FB-8ABF-B9F2C969F083 Figs 14E–14H, 15 and 16.

Examined material. Holotype: NHMO C6963. Al Ferdan, Suez Canal, 30° 40' 12.4'' N, 32° 20' 6.8' 'E. Paratypes (3 specimens): NHMO C6964, Eladabia, Gulf of Suez, 29° 56' 6.0'' N, 32° 28' 36.6'' E; NHMO C 6965, Eladabia, Gulf of Suez, 29° 56' 6.0'' N, 32° 28' 36.6'' E; NHMO C6966, off Alexandria (Mediterranean Sea), 31° 12' 43'' N, 29° 53' 2.4' 'E.

Re–description. Maxillary formula: I = 1+1, II = 4+4, III = 5+0, IV = 4+6, V = 2+1, VI absent (paratype NHMO C6964, Fig 15A and 15B); fully agrees with original description except in having: (1) anterior most parapodia with conical ventral cirri (instead of having inflated basis along whole body) (Fig 15C and 15D); (2) three black aciculae per parapodium (instead of four, three black and one yellow) (Figs 15E, 15F and 16A); (3) one (occasionally absent, occasionally two in posterior chaetigers) unidentate subacicular hook (instead of subacicular hooks absent, reported as “yellow acicula” in the original description) (Figs 15E, 15F, 16B and 16C), present from chaetiger 32 (paratype NHMO C6966), 40 (holotype, paratype NHMO C6964), 48 (paratype NHMO C6965); (4) two pairs of pygidial cirri, ventral ones absent but scars indicating presence (instead of lacking ventral pair of pygidial cirri); (5) pectinate chaetae of four types; Type 1 in anterior body region, progressively replaced at midbody by Type 2; Types 3 and 4 in posterior and posterior–most chaetigers, respectively (instead of three types, with Type 1 in anterior and midbody segments and Types 2 and 3 in posterior segments); (6) Type 1 thin, symmetrical, isodont with external teeth markedly differing in length, with ca. 10–15 teeth; Type 2 thin, asymmetrical, isodont, with > 25 teeth; Type 3 relatively thick, asymmetrical, isodont, with about 15 teeth, coarse, with pointed tips; Type 4 thick, asymmetrical, anodont, with about five long and coarse teeth (instead of Type 1 isodont, with about 19 teeth, Type 2 with ca. 9 teeth and Type 3 with six teeth); and (7) Type 4 with teeth length *vs*. tip width ratio of 1 and teeth length *vs*. width ratio of 4 (not mentioned in original description) (Figs 14E–14H and 16D–16G).

## Discussion

### Distribution range of *Marphysa sanguinea*

Robust taxonomic literature strongly supports not only that *M*. *sanguinea* fails to be a cosmopolitan species, but also that its distribution seems to be surprisingly restricted to areas surrounding its type locality [29, 30, 36]. Its current confirmed distribution ranges from Arcachon (Bay of Biscay, Atlantic coast of France) as southern limit, to the Southern Bight region (North Sea) as north-eastern limit [35, 36], including both shores of the English Channel (type locality). The species has also been recorded in nearby regions, such as the western Irish coast [94], the Irish and Welsh shores of the Celtic Sea [95–98], and the Bristol Channel and Severn Estuary (e.g., [95, 97–99] and references herein), in what could represent its north-western limit. Moreover, the Natural History Museum of UK (NHM) holds in its collections specimens identified as *M*. *sanguinea* from the Bristol Channel (NHMUK 1954.1.1.127, from Woody Bay; NHMUK 1970.7, from Porlock Weir). The species was also reported as “common” at Sully and Lavernock Point (Welsh coast of inner Bristol Channel) [99] and as “relatively rare” [98] or “occasional” [96] at Dale (Pembrokeshire, Welsh coasts of Celtic Sea), where it was not found in a 1988 survey [100]. Although we have not revised any of these records, many of them refer to intertidal specimens associated to hard substrates, so that at least some of them may feasibly represent the north-western limit of distribution for *M*. *sanguinea*.

The species has been recently reported as “introduced” in the southern North Sea, namely in southwestern Dutch shores [35, 101–103], where it is listed as an alien species. However, this statement seems to lack supporting evidence. Although there are not many published reports, *M*. *sanguinea* was previously known from the English coasts of the North Sea, at Whitstable, Kent [104] and Skipper’s Island, Essex [105] and, more recently, from a shipwreck off Oostende, Belgium [106]. Furhermore, the NHM holds in its collections specimens identified as *M*. *sanguinea* from Norfolk (NHMUK 1903.5.16.1), Burnham-on-Crouch, Essex (NHMUK 1966.10.4), and Margate Beach, Kent (NHMUK 1966.4.21). Although we have not revised the identity of these specimens, all them support the existence of populations of *Marphysa* in the Southern Bight region for more than one century. However, the scarce records in the Southern Bight and their absence in the northern regions of the North Sea (with one exception, see below) seem to indicate that the genus is, if not accidental, at least rare or occurring there at low densities. This supports the Southern Bight as being the north-eastern limit of distribution for the genus (including *M*. *sanguinea*). Consequently, we suggest that the relatively rare presence of *M*. *sanguinea* in the Netherlands is more likely connected with the natural limit of its distribution, rather than to a human mediated introduction [35], with the recent findings in the area more probably resulting from achieved newly routine and more exhaustive monitoring programs.

The single report of *Marphysa* in the northern regions of the North Sea corresponds to a specimen described (but not named) from the Swedish west coast (near Uddevalla, Skagerrak) [107], a region under climatic and oceanographic conditions very different from those prevailing in the English Channel and Southern Bight. This specimen differs clearly from the *sanguinea*–group by the presence of tridentate composite falcigers in the anterior 15 chaetigers (against the single presence of composite spinigers in the *sanguinea*–group), a very uncommon feature, if not unique, in the genus. Regardless whether it may represent a undescribed species, it clearly differs from all the species targeted in our work.

The low records of *M*. *sanguinea* in north European waters above the Southern Bight or the Celtic Sea seem to indicate temperature as a key limiting factor for the northern distribution of this intertidal species. This is in accordance with the distribution of other intertidal invertebrates along the English Channel coasts, which diminish their densities eastwards in the direction of the Strait of Dover), were not only the average water temperature is lower, but also the severe winter cold results in a reduced fitness for southern species, which are replaced by northern taxa [108]. Regarding the Celtic Sea and Bristol Channel side, local episodes of high mortality of *M*. *sanguinea* were registered at certain areas of south Wales after the 1962–63 severe winter [98]).

As for the southern limit of distribution of *M*. *sanguinea*, it still needs further investigation along the Atlantic coasts of France, Spain and Portugal. The present descriptions of *M*. *gaditana* sp. nov. and *M*. *chirigota* sp. nov. from the Bay of Cádiz place that southern limit somewhere between Arcachon and Cádiz. Very likely, it may be north of the Sado Estuary (Portugal), as our analyses place the GenBank sequence of one specimen from that locality within *M*. *gaditana* sp. nov.

### State of the art after type species resdescription

Since *M*. *sanguinea* redescription [29], two new species of the *sanguinea*–group were described from European and nearby locations, *M*. *victori* from the Bay of Biscay [3] and *M*. *aegypti* from the eastern Mediterranean, Suez Canal and Gulf of Suez [31], while we are here describing two more from the Bay of Cádiz. Similar situations occurred in other well studied coasts, such as Australia [29, 52], South Africa [30], the Grand Caribbean [77], China [33, 34] and Hong-Kong [51], where the presence of *M*. *sanguinea* proved to result from misidentifications or from a wrong use of the “cosmopolitan species” concept. Accordingly, several species (many new, some recovered from synonymies) have been reported, while others are still waiting to be reanalysed, likely to have their status removed from synonymy. Among them, *Marphysa haemasona* Quatrefages, 1866, (South Africa), *M*. *leidii* Quatrefages, 1866 (Atlantic USA), *M*. *parishii* Baird, 1869 (Brazil), *M*. *iwamushi* Izuka, 1907 (Japan), or *M*. *sanguinea americana* Monro, 1933 (Pacific Panama) [19, 23, 91, 109]. As a result, the so–called *sanguinea*–group [50] or Group–B [110] currently comprises 32 species or subspecies of *Marphysa*, including one recently described from Philippines [8] and the two new ones described herin. Certainly, many more wait to be discovered in the near future.

Another important aspect allowing recognising and delineating the species of *Marphysa* is the habitat. *Marphysa sanguinea* seems to be virtually always present in association with hard substrates [25, 111, 112], while most species of the *sanguinea*–group occur in different, likely species-specific substrates [36], usually soft. In the case of the two new species here described, this habitat specificity also applies, as *M*. *gaditana* sp. nov. was found in muddy substrates, while *M*. *chirigota* sp. nov. was associated to sediments with higher contents of the sand fraction.

In addition to highlighting habitat specificity, we would also like to stress two other main aspects that emerge as useful issues in providing solutions to this species group. First, the obvious growing molecular standards. In our case, we were able to state, with different degrees of certitude, that both *M*. *gaditana* sp. nov and *M*. *chirigota* sp. nov. differed from all previously sequenced species of the genus, but also that the former apparently occurs in a number of different locations at both sides of the Atlantic, namely France, Portugal and USA. Second, the increasingly careful and detailed observations leading to highlight the presence of clearly discriminatory characters, many of them previously overlooked. In the case of *Marphysa*, in addition to traditional morphological traits (e.g., the shape of dorsal cirri and pre–chaetal, chaetal and post–chaetal lobes, the starting chaetiger of brachiae or subacicuar hooks, or the type of compound chaetae [77, 81]), the shape of pectinate chaetae became a key argument, as first postulated for the type species [35] and later for some Chinese species of the *sanguinea*–group [34]. Classifying pectinate chaetae in a formulaic way may be tricky [8], but our results confirm the relevancy of carefully observing their morphology, number and presence, as well as the variations along the whole body. We also provide additional morphometric support based on the use of width/length ratios for the teeth of these chaetae in distinguishing among species, which turned to be particularly relevant when comparing *M*. *aegypti* and *M*. *chirigota* sp. nov.

### Commercial interests and associated risks

Many polychaetes have a great commercial interest, and the species of *Marphysa* are not an exception. Most of them are being widely used as fishing bait by anglers all around the world, which is particularly favoured by two facts: 1) many of them occur intertidally or shallow subtidally, often in sheltered coasts, being thus easily collectable by hand or by digging the sediment, and 2) their relatively big size and robust muscular body is particularly adequate to be used as fishing bait. Their stiff bodies enable a tight fixation to fishing hooks, from which they cannot be easily detached. They most often remain intact if bitten by small fishes, being thus available for a bigger catch, which improves size selection and capture outcomes (Nuno Lopes, personal communication, 28 April 2019). Some anglers also sustain that the specimens of *Marphysa* may be bioluminescent, which would make them particularly attractive for night fishing (Nuno Lopes, personal communication, 28 April 2019). However, this statement still requires scientific confirmation.

Endurance once in the hook and catch selectivity have thus made the species of *Marphysa* sought and popular fishing baits everywhere in the world for such a long time. Scientific records of this particular use are known from Australia [29, 35], China [33, 34], Egypt [31], England and the English Channel [25, 113], the French Atlantic [3] and Mediterranean coasts [21], Japan [23, 24, 26, 27], Malaysia [32, 114], South Africa [30], Sri Lanka [28], or Zanzibar [22]. However, our results emphasize the importance of knowing how many species are being currently traded under the name “*M*. *sanguinea*”, not only in the Iberian Peninsula, but also in Europe and all around the world [1, 13]. The fact that the south Iberian “*M*. *sanguinea*” turned to be two different, new species, as well as their differences in size, behaviour and habitat, indicate that they may have different life cycles, which probably also differ from those of the genuine *M*. *sanguinea* and any other species within the *sanguinea*–group. This may have obvious consequences for any commercial initiative (e.g., aquaculture, fishing baits), as well as for management programs of exploited natural populations, which may be extrapolated to all species within the group.

Our results also contribute to highlight the relevance and necessity of accurate taxonomic studies dealing with species of commercial interest. Exploited saleable species used to be distributed locally, while at present, the growing global marked establishes the potential of being distributed worldwide [1, 2, 13]. The usage of incorrect identifications favours careless practices, and enables impunity in trading, transporting and (possibly) releasing living allochthonous species into the wild. As they are being officially traded under the same scientific name than autochthonous species, no legal actions can be taken if a, let us say, “*M*. *sanguinea*” from a remote region of the globe is released, accidentally or not, in a Mediterranean area were “*M*. *sanguinea*” also occurs. This way, different exotic species may be legally introduced to areas where they are non–native. This may be the case of *M*. *gaditana* sp. nov. in some of the locations here reported (particularly in the most remote ones). It must be highlighted that, at least at Cap de la Hague (France), this species lives in sympatry with *M*. *sanguinea* (although they occupy different habitats, i.e., soft and hard bottoms, respectively). Sympatry has been also reported for other species of the *sanguinea* group in Philippines, Zanzibar, the Florida Keys, Australia and Cádiz Bay ([8], present work). The fact is that exotic species may perfectly survive to establish viable populations outside their native habitats after being released to the wild by anglers. This not only represents a risk due to the own presence of the introduced polychaete, but also may favour introductions of potentially dangerous associated organisms. A recent example has been reported for the beachworm *Perinereis linea* (Treadwell, 1936) [115], a traded fishing bait native from the NW Pacific that has a well–established populations in the Mar Menor lagoon (Mediterranean coast of the Iberian Peninsula) [116]. The reproductive females of this exotic species carry gelatinous egg masses where the embryos are attacked by symbiotic ciliate protozoans, thus keeping the potential of acting as carriers of diseases for the native beachworms [116].

An interesting additional question rises on whether all known species of the *sanguinea* group are native or introduced in the areas from where they were first described. Solving this question, however, would require having samples from all around the world to undertake a complex molecular analyses, which is completely out of the scope of our paper. Despite this, knowing the real identity of commercially exploited species may certainly contribute to recognise the risks and, thus, to control them by promoting the implementation of good habits among traders, but also among the possible final users (e.g., sport anglers). As introduced exotic species may always have the potential of becoming invasive, the consequences of lacking these controls for local species and habitats would be unpredictable, but certainly point to overall changes in biodiversity that would further affect food webs, ecosystem functioning, and the provision of ecosystem services [117].

### The collapsing “cosmopolitan species” concept

The use of polychaetes as model organisms in many different types of studies, from biogeochemistry, biology and physiology to ecology and genetics, as well as their commercial interest and increasing trade market, combines with old incomplete descriptions and inadequate diagnostic features to generate a considerable number of worldwide citations for certain species that obviously lack a rigorous taxonomic support. *Marphysa sanguinea* is a perfect example of this problem. This species has been reported in many studies from locations as diverse as Japan, China, Hong-Kong, South Korea, Australia, USA, Morocco, South Africa, India, New Caledonia, Gulf of Aden, Persian Gulf, Gulf of Thailand and many more (e.g.,[36, 118–121] and references therein), with examples of misidentifications all around the world existing mainly, but not exclusively, in faunistic or ecological papers. Misidentifications certainly include European waters, where specimens of supposedly *M*. *sanguinea* have been used to trace heavy metals and for diet tests based on survival and growth of juveniles in Portugal [122, 123], to monitor life cycles in the Venice Lagoon [124], or as target of potential interest for aquaculture in the Bay of Cádiz [16, 17]. As for other world citations, our results strongly support that many of these European reports do not refer to *M*. *sanguinea*, but to different species within the genus.

As a final remark, we would like to highlight that proving the incorrectness of the “cosmopolitan” character traditionally attributed to many polychaete species should no longer be considered a surprise or even an added value increasing the interest of a given research or publication. Virtually all “cosmopolitan” polychaete species that have been confronted with careful, detailed studies (morphological, molecular or, ideally, both combined) have shown geographically restricted distributions, habitat specificity and/or the existence of hidden species complexes [125]. As a result, most species having broad worldwide distributions would very probably be those that have been secondarily spread (i.e., introduced) by human activities.

## Supporting information

S1 File Species delimitation. 16S fragment: PTP results: based on the Maximum Likelihood and Bayesian inference reconstructions. 16S fragment: PTP results: based on the Maximum Likelihood reconstruction. Species described in this paper highlighted in red.
(DOCX)
