# Correction: Digging the diversity of Iberian bait worms *Marphysa* (Annelida, Eunicidae)

**DOI:** 10.1371/journal.pone.0233825

**Published:** 2020-05-21

**Authors:** Daniel Martin, João Gil, Joana Zanol, Miguel A. Meca, Rocío Pérez Portela

There are a number of errors in Table 1. The GenBank accession numbers for the COI sequences listed in the fourth column should be MN 816442, MN 816443, and MN816444 for *M*. *chirigota* and MN816441 for *M*. *gaditana*. Please see the complete, correct Table 1 here.

**Table 1:** Species and sequences included in the molecular analyses.

			GenBank Accession Number	
Family	Species	Locality	COI	16S rDNA
Eunicidae	*M*. *chirigota* sp. nov.	Cádiz Bay, SW Iberian Peninsula	MN816442	MN813670
			MN816443	MN813671
			MN816444	MN813672
	*M*. *gaditana* sp. nov.	Cádiz Bay, SW Iberian Peninsula	MN816441	MN813673
			Non available	MN813674
	*M*. *aegypti* Elgetany, El-Ghobashy, Ghoneim and Struck, 2018 [31]	Al ferdan, Suez Canal	MF196968 [31]	Non available
		off Alexandria, Mediterranean Sea	MF196969, MF196970, MF196971 [31]	Non available
	*M*. *bifurcata* Kott, 1951 [43]	Northeast Australia	KX172177, KX172178 [44]	Non available
	*M*. *brevitentaculata* Treadwell, 1921 [45]	Quintana Roo, México	GQ497548 [46]	GQ478158 [46]
	*M*. *californica* Moore, 1909 [47]	California, USA	GQ497552 [46]	GQ478162 [46]
	*M*. *corallina* (Kinberg, 1865) [48]	South Africa	KT823271, KT823300, KT823306, KT823343, KT823371, KT823389, KT823410 [49]	Non available
	*M*. *fauchaldi* Glasby & Hutchings, 2010 [50]	North Australia	KX172165 [44]	Non available
	*M*. *hongkongensa* Wang, Zhang & Qiu, 2018 [51]	Tolo Harbour, Hong Kong	MH598525 [51]	MH598527 [51]
			MH598526 [51]	MH598528 [51]
	*M*. *iloiloensis* Glasby, Mandario, Burghardt, Kupriyanova, Gunton & Hutchings, 2019 [8]	Philippines	MN133418, MN106279, MN106280, MN106281 [8]	Non available
	*M*. *kristiani* Zanol, da Silva & Hutchings, 2016 [52]	Southeast Australia	KX172141, KX172142, KX172143, KX172144, KX172145, KX172146, KX172147, KX172148, KX172149, KX172150, KX172151, KX172155, KX172152, KX172153, KX172154, KX172156, KX172157, KX172158, KX172159, KX172160, KX172161, KX172162, KX172163 [52]	Non available
	*M*. *mullawa* Hutchings & Karageorgopoulos, 2003 [29]	East and Southeast Australia	KX172166, KX172167, KX172168, KX172169, KX172170, KX172171, KX172172, KX172173, KX172174, KX172175, KX172176 [52]	Non available
	*M*. *sanguinea* (Montagu, 1813) [15]	Callot Island, Northern Bretagne, France(locality corrected from that in [46]	GQ497547 [46]	GQ478157 [46]
		? [53]	Non available	AY838836 [53]
		Cornwal, UK	MK541904, MK950851, MK950852 [36]	Non available
		Arcachon, France	MK950853 [36]	Non available
		Brest, France	MK967470 [36]MN106282, MN106283, MN106284 [8]	Non available
	*M*. *mossambica* (Peters, 1854) [54]	Philippines	JX559751 [46]	JX559747 [46]
		Australia	KX172164 [52]	Non available
	*M*. *pseudosessiloa* Zanol, da Silva & Hutchings, 2017 [52]	Southeast Australia	KY605405, KY605406 [44]	Non available
	*M*. *regalis* Verrill, 1900 [55]	Ceará, Brazil	GQ497562 [46]	GQ478165 [46]
	*M*. *tripectinata* Liu, Hutchings & Sun, 2017 [33]	Beihai, China	MN106271, MN10622, MN1062723, MN106274, MN106275, MN106276, MN106277, MN106278 [33]	Non available
	*M*. *victori* Lavesque, Daffe, Bonifácio & Hutchings, 2017 [3]	Arcachon Bay, France	MG384996, MG384999 [3]	Non available
			MG384997 [3]	MG385000 [3]
			MG384998 [3]	MG385001 [3]
	*M*. *viridis* Treadwell, 1917 [56]	Ceará, Brazil	GQ497553 [46]	GQ478163 [46]
	*Marphysa* sp.	Sado Estuary, Portugal	KR916870 [57]	Non available
		Cap de la Hague, France^1^	AY040708 [58]	Non available
		Sado Estuary, Portugal^1^	KR916871, KR916872, KR916873 [57]	Non available
		Eastern Shore, Virginia, USA^1^	KP254223, KP254644 KP254890, KP255196 [59]	Non available
		Indian River Lagoon, Florida, USA^1^	KP254503, KP254537, KP254643, KP254743, KP254802 [59]	Non available
		China^1^	NC023124* [60]	NC023124* [60]
		China^1^	KF733802* [60]	KF733802* [60]
	*Paucibranchia bellii* (Audouin & Milne Edwards, 1833) [61] ^2^	Bay of Biscay, Spain	KT307661 [62]	Non available
		Banyuls, France	Non available	DQ779623 [63]
		?	Non available	AY838835 [53]
	*Paucibranchia disjuncta* (Hartman, 1961) [64]^2^	California, USA	GQ497549 [46]	GQ478159 [46]
	*Paucibranchia* sp.^2^ [65]^3^	Philippines	JX559753 [66]	JX559750 [66]
	*Nicidion angeli* (Carrera-Parra and Salazar-Vallejo, 1998) [67]^2^	Carrie Bow Cay, Belize	Non available	GQ478161 [46]
	*Palola viridis* Gray *in* Stair, 1847 [68]	Kosrae, Micronesia	GQ497556 [46]	GQ478167 [46]
	*Eunice* cf. *violaceomaculata* Ehlers, 1887 [69]	Carrie Bow Cay, Belize	GQ497542 [46]	GQ478148 [46]
	*Leodice rubra* (Grube, 1856) [70]	Ceará, Brazil	GQ497528 [46]	GQ478132 [46]
Onuphidae	*Hyalinoecia* sp.	Massachusetts, USA	GQ497524 [46]	GQ478125 [46]

*Complete mitochondrial genome.

^1^Species identified as *M*. *sanguinea* in GenBank, but identification incorrect according to [3] and our study.

^2^Genus updated, species is under *Marphysa* in GenBank.

^3^This specimen belongs to the *Marphysa bellii* group (JZ, personal observation). This group was recently considered to represent a different genus and described as *Paucibranchia*.
