# Supplementary material for: Digging the diversity of Iberian bait worms Marphysa (Annelida, Eunicidae)
Source: PLoS One. 2020 Jan 22;15(1):e0226749. doi: 10.1371/journal.pone.0226749 (PMC6975537; doi:10.1371/journal.pone.0226749)
Supplement: S1 File — 16S fragment: PTP results: based on the Maximum Likelihood and Bayesian inference reconstructions. 16S fragment: PTP results: based on the Maximum Likelihood reconstruction. Species described in this paper highlighted in red. (DOCX) [file pone.0226749.s001.docx]

**Species delimitation- 16S fragment: PTP results**

**Results based on the Maximum Likelihood reconstruction**

**Maximum Likelihood partition**

Species 1 (support = 1.000)

GQ478165

Species 2 (support = 1.000)

AY838835 *Marphysa bellii*

Species 3 (support = 1.000)

DQ779623 *Marphysa bellii*

Species 4 (support = 1.000)

GQ478159

Species 5 (support = 1.000)

JX559750

Species 6 (support = 1.000)

JX559747 *Marphysa mossambica*

Species 7 (support = 0.994)

MH598528 *Marphysa hongkongensa*,MH598527 *Marphysa hongkongensa*

Species 8 (support = 1.000)

GQ478163

Species 9 (support = 0.969)

**MN813670** ***Marphysa chirigota sp. nov.*_1, MN813671** ***Marphysa chirigota sp. nov.* _2, MN813672** ***Marphysa chirigota sp. nov.* _3**

Species 10 (support = 1.000)

GQ478162

Species 11 (support = 1.000)

GQ478158

Species 12 (support = 0.998)

**MN813673** ***Marphysa gaditana sp. nov.*_4, MN813674** ***Marphysa gaditana sp. nov.* _5**

Species 13 (support = 0.987)

KF733802,NC023124 *Marphysa sanguinea*

Species 14 (support = 0.991)

MG385001 *Marphysa victori*,MG385000 *Marphysa victori*

Species 15 (support = 1.000)

GQ478157

Species 16 (support = 1.000)

AY838836 *Marphysa sanguinea*

**Results based on the Bayesian inference reconstruction**

Species 1 (support = 1.000)

GQ478165

Species 2 (support = 1.000)

AY838835 *Marphysa bellii*

Species 3 (support = 1.000)

DQ779623 *Marphysa bellii*

Species 4 (support = 1.000)

GQ478159

Species 5 (support = 1.000)

JX559750

Species 6 (support = 1.000)

JX559747 *Marphysa bellii*

Species 7 (support = 0.994)

MH598528 *Marphysa hongkongensa*,MH598527 *Marphysa hongkongensa*

Species 8 (support = 1.000)

GQ478163

Species 9 (support = 0.969)

**MN813670** ***Marphysa chirigota sp. nov.*_1, MN813671** ***Marphysa chirigota sp. nov.* _2, MN813672** ***Marphysa chirigota sp. nov.* _3**

Species 10 (support = 1.000)

GQ478162

Species 11 (support = 1.000)

GQ478158

Species 12 (support = 0.998)

**MN813673** ***Marphysa gaditana sp. nov.*_4, MN813674** ***Marphysa gaditana sp. nov.* _5**

Species 13 (support = 0.987)

KF733802, NC023124 *Marphysa sanguinea*

Species 14 (support = 0.991)

MG385001 *Marphysa victori*,MG385000 *Marphysa victori*

Species 15 (support = 1.000)

GQ478157

Species 16 (support = 1.000)

AY838836 *Marphysa sanguinea*

**Species delimitation- COI fragment: PTP results**

**Results based on the Maximum Likelihood reconstruction**

**Maximum Likilhood partition**

Species 1 (support = 1.000)

JX559753 *Marphysa* sp.

Species 2 (support = 1.000)

GQ497562 *Marphysa regalis*

Species 3 (support = 0.940)

KT823410 *Marphysa corallina*, KT823371 *Marphysa corallina*, KT823389 *Marphysa corallina*, KT823343 *Marphysa corallina*, KT823306 *Marphysa corallina*, KT823300 *Marphysa corallina*, KT823271 *Marphysa corallina*

Species 4 (support = 1.000)

GQ497553 *Marphysa viridis*

Species 5 (support = 1.000)

KX172165 *Marphysa fauchaldi*

Species 6 (support = 0.991)

KX172164 *Marphysa mossambica*, JX559751 *Marphysa mossambica*

Species 7 (support = 0.575)

KX172163 *Marphysa kristiani, K*X172162 *Marphysa kristiani, K*X172161 *Marphysa kristiani, K*X172160 *Marphysa kristiani, K*X172159 *Marphysa kristiani, K*X172158 *Marphysa kristiani, K*X172157 *Marphysa kristiani, K*X172156 *Marphysa kristiani, K*X172155 *Marphysa kristiani, K*X172154Mristiani, KX172153 *Marphysa kristiani, K*X172152 *Marphysa kristiani, K*X172151 *Marphysa kristiani, K*X172150 *Marphysa kristiani, K*X172148 *Marphysa kristiani, K*X172147 *Marphysa kristiani, K*X172145 *Marphysa kristiani, K*X172144 *Marphysa kristiani, K*X172143 *Marphysa kristiani, K*X172142 *Marphysa kristiani, K*X172141 *Marphysa kristiani, K*X172149 *Marphysa kristiani, K*X172146 *Marphysa kristiani*

Species 8 (support = 1.000)

GQ497552 *Marphysa californica*

Species 9 (support = 0.994)

KX172177 *Marphysa bifurcata*, KX172178 *Marphysa bifurcata*

Species 10 (support = 0.997)

KY605405 *Marphysa pseudosessiloa*, KY605406 *Marphysa pseudosessiloa*

Species 11 (support = 1.000)

GQ497548 *Marphysa brevitentaculata*

Species 12 (support = 0.940)

KX172166 *Marphysa mullawa*, KX172176 *Marphysa mullawa*, KX172175 *Marphysa mullawa*, KX172174 *Marphysa mullawa*, KX172168 *Marphysa mullawa*, KX172167 *Marphysa mullawa*, KX172173 *Marphysa mullawa*, KX172172 *Marphysa mullawa*, KX172171 *Marphysa mullawa*, KX172169 *Marphysa mullawa*, KX172170 *Marphysa mullawa.*

Species 13 (support = 0.947)

MG384999 *Marphysa victori*,MG384997 *Marphysa victori*, MG384998 *Marphysa victori*, MG384996 *Marphysa victori*

Species 14 (support = 0.992)

KF733802  *Marphysa sanguinea,* NC023124Msanguinea

Species 15 (support = 0.953)

**MN816444 *Marphysa gaditana sp. nov.*_4,** KR916872 *Marphysa sanguinea*, KR916873 *Marphysa sanguinea*, KP255196 *Marphysa sanguinea*, KP254890 *Marphysa sanguinea*, KP254644 *Marphysa sanguinea*, KP254223 *Marphysa sanguinea*, KP254802 *Marphysa sanguinea*, KP254743 *Marphysa sanguinea*, KP254643 *Marphysa sanguinea*, KP254503G *Marphysa sanguinea*, KP254537 *Marphysa sanguinea*, KR916871 *Marphysa sanguinea*, AY040708 *Marphysa sanguinea*

Species 16 (support = 0.960)

MH598525 *Marphysa hongkongensa*,MH598526 *Marphysa hongkongensa*

Species 17 (support = 0.946)

AMW50118 *Marphysa iloiloensis*,AMW50120 *Marphysa iloiloensis*, AMW50122 *Marphysa iloiloensis*

Species 18 (support = 1.000)

GQ497549 *Marphysa* disjuncta

Species 19 (support = 1.000)

KT307661 *Marphysa bellii*

Species 20 (support = 0.953)

AMW50108 *Marphysa tripectinata*, AMW50109 *Marphysa tripectinata*, AMW50110 *Marphysa tripectinata*,AMW50111 *Marphysa tripectinata*, AMW50112 *Marphysa tripectinata*, AMW50113 *Marphysa tripectinata*, AMW50114 *Marphysa tripectinata*, AMW50115 *Marphysa tripectinata*

Species 21 (support = 0.956)

GQ497547 *Marphysa sanguinea*, MK967470 *Marphysa sanguinea*, MK541904 *Marphysa sanguinea*, MK950852 *Marphysa sanguinea*, MK950853 *Marphysa sanguinea*, MK950851 *Marphysa sanguinea*, AMW49086 *Marphysa sanguinea*, AMW49087 *Marphysa sanguinea*, AMW49088 *Marphysa sanguinea*

Species 22 (support = 1.000)

KR916870 *Marphysa* sp

Species 23 (support = 0.932)

MF196969 *Marphysa aegypti*, MF196971 *Marphysa aegypti*, MF196970 *Marphysa aegypti*, MF196968 *Marphysa aegypti*

Species 24 (support = 0.851)

**MN816441 *Marphysa chirigota sp. nov.*_1, MN816442 *Marphysa chirigota sp. nov.* _2, MN816443 *Marphysa chirigota sp. nov.* _3**

**Most supported partition found by simple heuristic search**

Species 1 (support = 1.000)

JX559753 *Marphysa* sp

Species 2 (support = 1.000)

GQ497562 *Marphysa regalis*

Species 3 (support = 0.940)

KT823410 *Marphysa corallina*, KT823371 *Marphysa corallina*, KT823389 *Marphysa corallina*, KT823343 *Marphysa corallina*, KT823306 *Marphysa corallina*, KT823300 *Marphysa corallina*, KT823271 *Marphysa corallina*

Species 4 (support = 1.000)

GQ497553 *Marphysa* viridis

Species 5 (support = 1.000)

KX172165 *Marphysa fauchaldi*

Species 6 (support = 0.991)

KX172164 *Marphysa mossambica*, JX559751 *Marphysa mossambica*

Species 7 (support = 0.575)

KX172163 *Marphysa kristiani, K*X172162 *Marphysa kristiani, K*X172161 *Marphysa kristiani, K*X172160 *Marphysa kristiani, K*X172159 *Marphysa kristiani, K*X172158 *Marphysa kristiani, K*X172157 *Marphysa kristiani, K*X172156 *Marphysa kristiani, K*X172155 *Marphysa kristiani, K*X172154Mristiani, KX172153 *Marphysa kristiani, K*X172152 *Marphysa kristiani, K*X172151 *Marphysa kristiani, K*X172150 *Marphysa kristiani, K*X172148 *Marphysa kristiani, K*X172147 *Marphysa kristiani, K*X172145 *Marphysa kristiani, K*X172144 *Marphysa kristiani, K*X172143 *Marphysa kristiani, K*X172142 *Marphysa kristiani, K*X172141 *Marphysa kristiani, K*X172149 *Marphysa kristiani, K*X172146 *Marphysa kristiani*

Species 8 (support = 1.000)

GQ497552 *Marphysa californica*

Species 9 (support = 0.994)

KX172177 *Marphysa bifurcata*, KX172178 *Marphysa bifurcata*

Species 10 (support = 0.997)

KY605405 *Marphysa pseudosessiloa*, KY605406 *Marphysa pseudosessiloa*

Species 11 (support = 1.000)

GQ497548 *Marphysa*

Species 12 (support = 0.940)

KX172166 *Marphysa mullawa*, KX172176 *Marphysa mullawa*, KX172175 *Marphysa mullawa*, KX172174 *Marphysa mullawa*, KX172168 *Marphysa mullawa*, KX172167 *Marphysa mullawa*, KX172173 *Marphysa mullawa*, KX172172 *Marphysa mullawa*, KX172171 *Marphysa mullawa*, KX172169 *Marphysa mullawa*, KX172170 *Marphysa mullawa*

Species 13 (support = 0.947)

MG384999 *Marphysa victori*, MG384997 *Marphysa victori*, MG384998 *Marphysa victori*, MG384996 *Marphysa victori*

Species 14 (support = 0.992)

KF733802 *Marphysa sanguinea,* NC023124Msanguinea

Species 15 (support = 0.953)

**MN816444 *Marphysa gaditana sp. nov.*_4,** KR916872 *Marphysa sanguinea,* KR916873 *Marphysa sanguinea,* KP255196 *Marphysa sanguinea*, KP254890 *Marphysa sanguinea*, KP254644 *Marphysa sanguinea*, KP254223 *Marphysa sanguinea*, KP254802 *Marphysa sanguinea*, KP254743 *Marphysa sanguinea*, KP254643 *Marphysa sanguinea*, KP254503G *Marphysa sanguinea*, KP254537 *Marphysa sanguinea*, KR916871 *Marphysa sanguinea,* AY040708Msanguinea

Species 16 (support = 0.960)

MH598525 *Marphysa hongkongensa*,MH598526 *Marphysa hongkongensa*

Species 17 (support = 0.946)

AMW50118 *Marphysa iloiloensis*, AMW50120 *Marphysa iloiloensis*, AMW50122 *Marphysa iloiloensis*

Species 18 (support = 1.000)

GQ497549 *Marphysa disjuncta*

Species 19 (support = 1.000)

KT307661 *Marphysa bellii*

Species 20 (support = 0.953)

AMW50108 *Marphysa tripectinata,* AMW50109 *Marphysa tripectinata,* AMW50110 *Marphysa tripectinata,* AMW50111 *Marphysa tripectinata,* AMW50112 *Marphysa tripectinata,* AMW50113 *Marphysa tripectinata,* AMW50114 *Marphysa tripectinata,* AMW50115 *Marphysa tripectinata*

Species 21 (support = 0.956)

GQ497547 *Marphysa sanguinea*, MK967470 *Marphysa sanguinea,* MK541904 *Marphysa sanguinea,* MK950852 *Marphysa sanguinea,* MK950853 *Marphysa sanguinea,* MK950851 *Marphysa sanguinea,* AMW49086 *Marphysa sanguinea,* AMW49087 *Marphysa sanguinea,* AMW49088 *Marphysa sanguinea*

Species 22 (support = 1.000)

KR916870 *Marphysa* sp

Species 23 (support = 0.932)

MF196969 *Marphysa aegypti*, MF196971 *Marphysa aegypti*, MF196970 *Marphysa aegypti*, MF196968 *Marphysa aegypti*,

Species 24 (support = 0.851)

**MN816441 *Marphysa chirigota sp. nov.*_1, MN816442 *Marphysa chirigota sp. nov.* _2, MN816443 *Marphysa chirigota sp. nov.* _3**
